# CF-PPiD technology based on cell-free protein array and proximity biotinylation enzyme for in vitro direct interactome analysis

**DOI:** 10.1038/s41598-022-14872-w

**Published:** 2022-06-22

**Authors:** Shusei Sugiyama, Kohdai Yamada, Miwako Denda, Satoshi Yamanaka, Satoshi Ozawa, Ryo Morishita, Tatsuya Sawasaki

**Affiliations:** 1 CellFree Sciences. Co. Ltd., 3 Bunkyo-cho, Matsuyama, Ehime 790-8577 Japan; 2 Proteo-Science Center, 3 Bunkyo-cho, Matsuyama, Ehime 790-8577 Japan

**Keywords:** Protein-protein interaction networks, Proteome

## Abstract

Protein–protein interaction (PPI) analysis is a key process to understand protein functions. Recently, we constructed a human protein array (20 K human protein beads array) consisting of 19,712 recombinant human proteins produced by a wheat cell-free protein production system. Here, we developed a cell-free protein array technology for proximity biotinylation-based PPI identification (CF-PPiD). The proximity biotinylation enzyme AirID-fused TP53 and -IκBα proteins each biotinylated specific interacting proteins on a 1536-well magnetic plate. In addition, AirID-fused cereblon was shown to have drug-inducible PPIs using CF-PPiD. Using the human protein beads array with AirID-IκBα, 132 proteins were biotinylated, and then selected clones showed these biological interactions in cells. Although ZBTB9 was not immunoprecipitated, it was highly biotinylated by AirID-IκBα, suggesting that this system detected weak interactions. These results indicated that CF-PPiD is useful for the biochemical identification of directly interacting proteins.

## Introduction

Many proteins form complexes with other proteins to properly function in cells. Analysis of protein–protein interactions (PPIs) therefore is a key process for understanding the biological functions of a target protein. Several different technologies have been used to identify partner proteins, such as the yeast two-hybrid system^1,2^, mass spectrometry analysis after immunoprecipitation^3,4^, and cell-free protein arrays, which we have previously described^5,6^. Many critical partner proteins have been found by these methods. Because intracellular proteins are regulated by complicated systems, such as signalling transduction cascades, using multiple different technologies can increase our understanding of cellular protein regulation.

Proximity-labelling technology using *Escherichia coli* biotin ligase BirA mutants has been widely used to identify partner proteins in cells and organisms^7–9^. An example of this technology is the BioID (proximity-dependent biotin identification) method^10^. In general, the BioID enzyme has promiscuous activity and releases highly reactive and short-lived biotinoyl-5′-AMP. The released biotinoyl-5′-AMP modifies proximal proteins (within 10 nm)^11^. BioID is used by expressing a BioID-fused protein and adding biotin. In cells or organisms expressing the BioID-fused target protein, proteins with which the target protein interacts are biotinylated and can be comprehensively analysed using streptavidin bead enrichment followed by mass spectrometry^8^. Recently, highly active proximity-labelling enzymes, such as TurboID^12^ and AirID^13^, have been reported that can be used to improve the BioID technology. However, the use of BioID technology is currently limited to the screening of partner proteins in cells or organisms.

Rapamycin binds to FK-binding protein 12 (FKBP12) and induces the interaction of FKBP12–Rapamycin complex to mTORC1, resulting in inhibition of mTORC1^14^. In plants, it has been reported that the main function of major phytohormones, such as auxin and jasmonic acid, is to induce PPIs between hormone receptors and regulators^15^. Furthermore, the main function of thalidomide and derivatives, which are used as drugs to treat multiple myeloma^16^, is to induce PPIs between the thalidomide binder cereblon (CRBN) and neo-substrates^17–19^. Thus, PPIs induced by chemical compounds are key interactions for the regulation of proteins.

Human protein arrays have been reported by several groups, including our group^20,21^. In our previous study, a human protein array consisting of 19,712 recombinant proteins was constructed by the combination of a wheat cell-free protein production system and a full-length human cDNA set^21^. Multiple-well plate or glass-slide formats were mainly used as the screening devices for the protein array^22,23^. Recently, we have developed a strong magnetic plate for capturing recombinant proteins and showed that the plate can be used for the validation of antibody specificity^21^. In the present study, we used this magnetic plate to develop a biochemical screening system for PPI analysis by combining BioID technology with a human protein array, which we termed cell-free human protein array technology and proximity biotinylation-based PPI identification (CF-PPiD). The proximity biotinylation enzyme AirID that we have reported previously^13^ was *N*-terminally fused to a target protein, such as p53 (TP53), CRBN, or IκBα. The fusion proteins were synthesized by the wheat cell-free system and directly used as targets without purification for the screening of interacting proteins on a protein array. The results indicated that a single AirID-fused target protein carried out normal PPI- and molecular glue- or proteolysis-targeting chimera (PROTAC)-dependent biotinylations. For large scale screening, 19,712 human proteins with *N*-terminal fused FLAG-glutathione-*S*-transferase (GST) were synthesized using the wheat cell-free system and subsequently were captured as dual spots on 27 1536-well formatted magnetic plates for construction of the protein array (20 K Human Protein Beads Array). The AirID-IκBα fusion protein was mixed with biotin on the plate for PPI screening. After vigorous washing, 132 biotinylated proteins showed a high biotinylation signal. Selected clones from these candidate partner proteins were biotinylated in cells. These results suggested that this CF-PPiD system is a useful biochemical screening approach for the genome-wide identification of direct partner proteins.

## Results

### Biotinylation of interacting proteins by AirID-fused proteins using the CF-PPiD system

**Figure 1:**
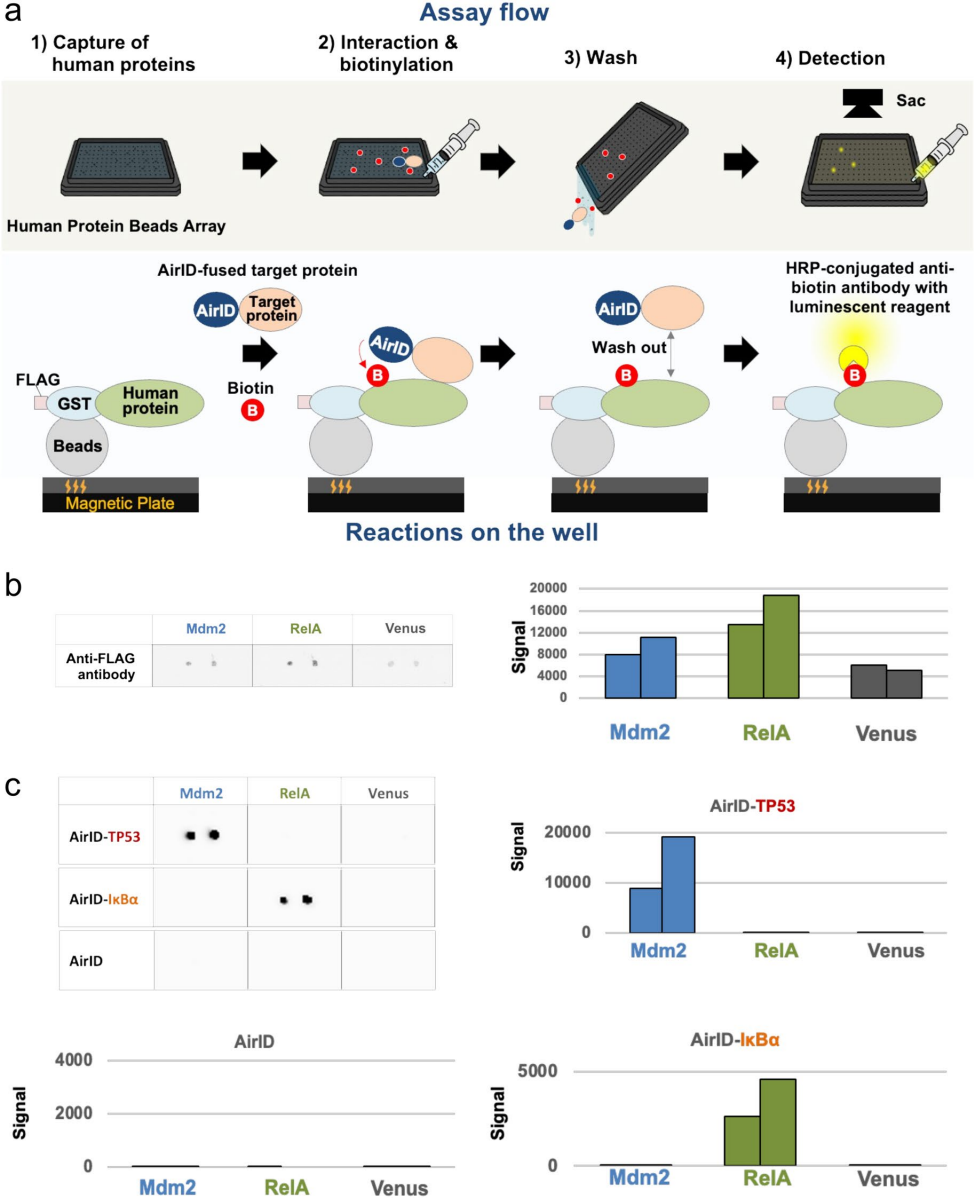
Biotinylation of interacting proteins by AirID-fused proteins using the CF-PPiD system. (**a**) General assay flow of CF-PPiD. The lower panel shows the reactions on the well. First, the human protein beads array is prepared. Human proteins are captured on each well of the array. Next, the AirID-fused target protein, biotin, and ATP are added to the array. The target protein interacts with partner proteins, and then AirID biotinylates the partner proteins in close proximity. Then, the reaction mixture, containing the AirID-fused target protein, is washed out. Finally, the biotin molecules on the partner proteins are detected by chemiluminescence using HRP-conjugated anti-biotin antibody. (**b**) Three FG-proteins (Mdm2, RelA, and Venus) were confirmed to be captured on the array using a fluorescently labelled anti-FLAG antibody. (**c**) Three AirID-fused proteins (AirID-TP53, AirID-IκBα, and AirID alone) and three FG-proteins (Mdm2, RelA, and Venus) captured on the magnetic plate were used as a model system for CF-PPiD. AirID-TP53 and AirID-IκBα specifically biotinylated FG- Mdm2 and FG-RelA, respectively. The bar graph shows the signal intensity. Source data are provided as a Source data file.

In the antibody validation system, CF-PA^2^Vtech, the human recombinant proteins for protein array were synthesized as FLAG-GST fusion proteins (FG-fusion proteins)^21^. In the same study, a magnetic plate device was developed to capture these proteins by glutathione-conjugated magnetic beads via an *N*-terminal fused GST protein. In the present study, the same magnetic protein capture system was used. The CF-PPiD system consists of four steps (Fig. 1a): (1) capture of a human protein via beads on a magnetic plate; (2) interaction with biotinylation; (3) washing by simple decantation; and (4) detection by horseradish peroxidase (HRP)-conjugated anti-biotin antibody. To validate the proximity biotinylation on the magnetic plate device, we used the two PPI models, IκBα–RelA and TP53–Mdm2, because the interactions between these two pairs of proteins are well known^24–26^. To check the performance of the CF-PPiD system, we investigated PPI-dependent biotinylation on the magnetic plate to detect interactions in the model systems. AirID *N*-terminally fused to TP53 (AirID-TP53) or IκBα (AirID-IκBα) and FG-tagged proteins (FG-Mdm2, FG-RelA, and FG-Venus) were synthesized by wheat cell-free protein production system (Supplementary Fig. 1a), and directly used for the assay. In a tube as the standard biochemical assay, AirID-TP53 biotinylated FG-Mdm2 but not FG-Venus (left panel in Supplementary Fig. 1b), and AirID-IκBα clearly biotinylated FG-RelA (right panel). For CF-PPiD, three substrate proteins were spotted on the magnetic plate, and subsequently were confirmed to be captured on the plate using an anti-FLAG antibody (Fig. 1b). The scanning values also indicated similar capture values between the proteins (right panel), indicating that this spotting method was sufficient to detect proteins. Next, a translational mixture containing AirID alone, or each AirID-fused protein (AirID-TP53 and AirID-IκBα), with biotin and ATP was applied to the magnetic plate by a 10-mL syringe. During the incubation, interacting proteins were biotinylated by AirID. Subsequently, the detection of biotinylated proteins was performed using an HRP-conjugated anti-biotin antibody with luminescent reagent. Using this system, AirID-TP53 and AirID-IκBα specifically biotinylated FG-Mdm2 and FG-RelA, respectively (Fig. 1c), whereas biotinylation was not observed for Venus and no interactions were observed between TP53 and RelA, or IκBα and Mdm2. The luminescent signal from biotinylated dual spots showed strong signals between both TP53–Mdm2 and IκBα–RelA (bar graphs in Fig. 1c). In contrast, AirID alone did not biotinylate the proteins. These results indicated that this system can be used to detect interacting proteins through biotinylation on the plate.

### Detection of molecular glue or PROTAC-dependent interactions by AirID-CRBN fusion protein with the CF-PPiD system

**Figure 2:**
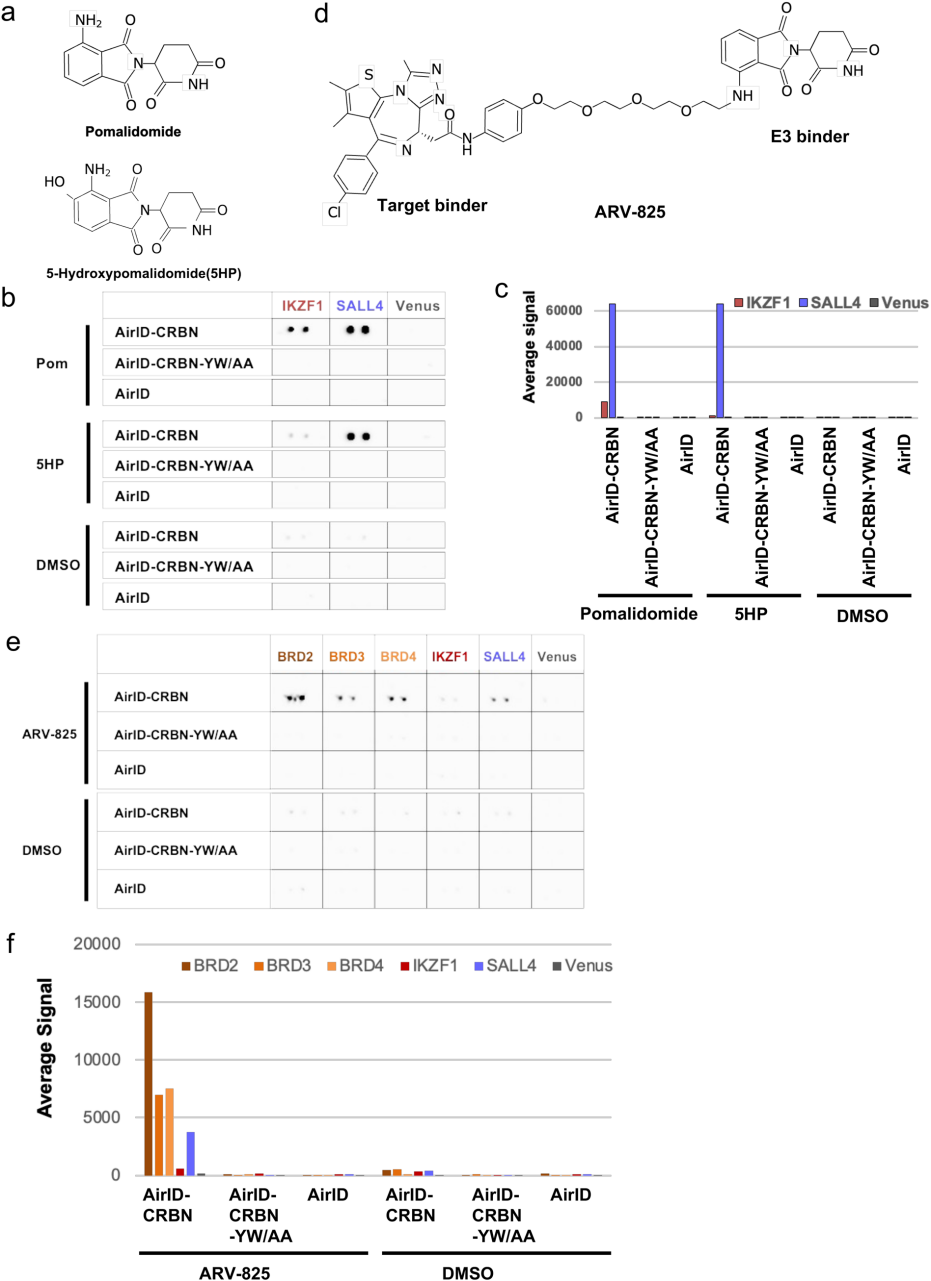
Detection of molecular glue- or PROTAC-induced biotinylation using the CF-PPiD system. (**a**) The chemical structures of pomalidomide and 5-hydroxypomalidomide (5HP). (**b**) To detect molecular glue-dependent PPIs, three AirID-fused proteins (AirID-CRBN, AirID-CRBN-YW/AA, and AirID) and three FG-proteins (IKZF1, SALL4, and Venus) captured on the magnetic plate were used for CF-PPiD with pomalidomide or 5HP. AirID-CRBN with pomalidomide specifically biotinylated IKZF1 and SALL4. With 5HP, instead of pomalidomide, only SALL4 was biotinylated. AirID alone and AirID-CRBN-YW/AA with pomalidomide or 5HP did not interact and biotinylate any proteins. AirID-CRBN with DMSO also did not biotinylate any proteins. (**c**) The bar graph shows the average signal intensity of the dual spots in (**b**). (**d**) Chemical structure of ARV-825. ARV-825 consists of a target binder and an E3 binder. (**e**) To detect PROTAC-dependent PPIs, three AirID-fused proteins (AirID-CRBN, AirID-CRBN-YW/AA, and AirID alone) and six FG-proteins (BRD2, BRD3, BRD4, IKZF1, SALL4, and Venus) captured on the magnetic plate were used for CF-PPiD with ARV-825. AirID-CRBN with ARV-825 biotinylated BRD2, BRD3, BRD3, IKZF1, and SALL4. Neither AirID alone, nor AirID-CRBN-YW/AA, with ARV-825, biotinylated any of these proteins. AirID-CRBN with DMSO also did not biotinylate any of the proteins. (**f**) The bar graph shows the average signal intensity of the dual spots in (**e**). Source data are provided as a Source data file.

Thalidomide and derivatives, such as lenalidomide and pomalidomide (upper panel in Fig. 2a), induce PPIs between CRBN and neo-substrates^17–19^. These compounds have been called a molecular glue because they attach two proteins together like a glue. We investigated whether this system can detect molecular glue-dependent PPIs via biotinylation. A PPI between CRBN and SALL4 or IKZF1 is strongly induced by pomalidomide in vitro and in cells^20,21^. In contrast, 5-hydroxypomalidomide (5HP, lower panel in Fig. 2a) has a low ability to induce a PPI between CRBN and IKZF1^27^. FG-IKZF1, FG-SALL4, and FG-Venus proteins were captured on the magnetic plate, and then AirID-CRBN fusion protein (AirID-CRBN) was added with pomalidomide or 5HP. The CRBN-Y384A/W386A mutant (AirID-CRBN-YW/AA) was also used as a negative control because this mutant cannot bind to thalidomide or its derivatives^28^. Using the CF-PPiD system with molecular glue (Supplementary Fig. 2a), AirID-CRBN with pomalidomide induced the biotinylation of FG-IKZF1 and FG-SALL4, and with 5HP, FG-SALL4 was biotinylated but not FG-IKZF1. AirID alone and the AirID-CRBN mutant did not biotinylate any proteins (Fig. 2b). The scan data also clearly showed that these biotinylations had a high signal value (right bar graph). These results indicated that this system could detect a molecular glue-dependent interaction with drug selectivity on the plate.

Recently, PROTAC, which consists of a target binder and E3 binder (Fig. 2c), has been developed as a new protein degrader^29^. Thalidomide or thalidomide derivatives are used as the E3 binder in many PROTACs^30,31^. PROTAC induces PPIs, similar to a molecular glue, between two proteins; the target and E3 ubiquitin ligase proteins^30,31^. For instance, ARV-825, a well-known PROTAC, which consists of the bromodomain (BRD) protein binder OTX-015 and pomalidomide (Fig. 2c), induces a PPI between BRD proteins and CRBN. To validate whether the CF-PPiD system can detect PROTAC-dependent PPIs, FG-BRD2, FG-BRD3, and FG-BRD4 proteins were added to the protein set described above (Fig. 2d), and ARV-825 was used as a model PROTAC. Using this system, AirID-CRBN with ARV-825 clearly induced the biotinylation of BRD2, BRD3, BRD4, IKZF1, and SALL4 (Fig. 2e), whereas the CRBN mutant (AirID-CRBN-YW/AA), or AirID alone, with ARV-825 or DMSO treatment did not induce biotinylation. The scan data clearly showed that these biotinylations had a high signal value (lower bar graph in Fig. 2f). These results indicated that the CF-PPiD system can detect molecular glue- and PROTAC-dependent PPIs with drug selectivity.

### Validation of the CF-PPiD system using a diversity protein array

**Figure 3:**
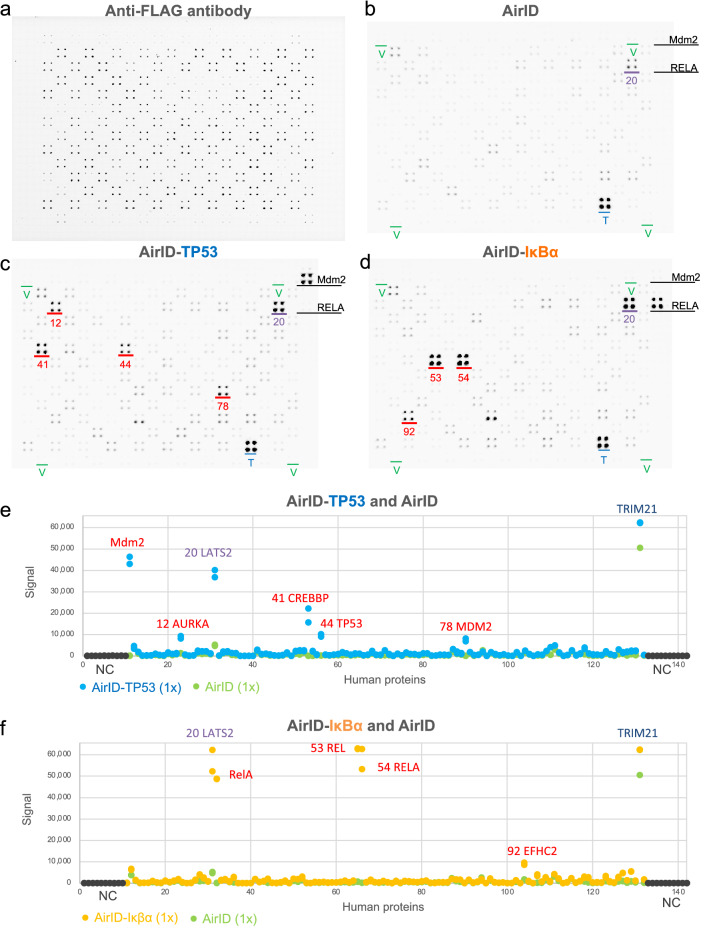
Validation of CF-PPiD using a diversity protein array. (**a**) The identity of the FLAG-GST tagged proteins spotted on the diversity protein array was confirmed using a fluorescently labelled anti-FLAG antibody. (**b**) The image of the PPI screening between AirID and the proteins on the diversity protein array. (**c**) The image of the PPI screening between AirID-TP53 and the proteins on the diversity protein array. (**d**) The image of the PPI screening between AirID-IκBα and the proteins on the diversity protein array. (**e**) The dot-blotting graph from scan data of the PPI screening using AirID or AirID-TP53 protein. (**f**) The dot-blotting graph from scan data of the PPI screening using AirID or AirID-IκBα fusion protein. The dots represent the intensity of 1 × beads spots. Positive spots are labelled in red. The spots of TRIM21, which interacted with antibody IgG, are labelled in dark blue. The spots of LATS2, which interacted with AirID alone, are labelled in purple. The numbers on the figures a-d indicate a clone No. of the spots. Green character V indicates Venus spots. Source data are provided as a Source data file.

In the small-scale assays described above, the CF-PPiD system detected PPIs via biotinylation (Figs. 1 and 2). We next planned to expand the number of proteins and to validate a wide variety of proteins on a 1536-well formatted plate. To make a middle-scale protein array, we selected 118 proteins, including protein kinases, transcription factors, and E3 ubiquitin ligases (Diversity Protein Array, Supplementary Fig. 3a and Supplementary Table 1), and arrayed each family of proteins on the plate (Supplementary Fig. 3b). To determine the detection levels of the captured proteins, we used double dual spots (four spots for each protein) having different protein amounts, which consisted of upper (1 × , approximately 0.015 µL of beads) and lower (3 × , approximately 0.045 µL of beads) dual spots. The proteins were spotted, grouped by protein function (Supplementary Fig. 3b). To investigate the detection range of the spotted proteins, we used an anti-FLAG antibody. Using the antibody confirmed that each protein was spotted on the magnetic plate, and the signals of the 118 spotted proteins ranged between approximately 60,000 [maximum clone No. 29 (spot No. 41)] and 3,264 [minimum clone No. 26 (spot No. 38)], within a 20-fold difference (Fig. 3a and Supplementary Fig. 3c). Furthermore, the scan data indicated that each signal from a 1 × spotted protein had a similar value to the corresponding 3 × protein. These results indicated that the 1 × protein concentration in the 1536-well format was sufficient for the detection of interacting proteins.

For validation using the diversity protein array, the three proteins AirID, AirID-TP53, and AirID-IκBα were used as target proteins. Because TRIM21 binds to the IgG protein^21,32^, TRIM21 resulted in a high non-specific signal in all arrays using IgG (blue character T in Fig. 3a–d and TRIM21 in Fig. 3e, f and Supplementary Fig. 4a–c). For AirID alone, almost all the clones resulted in a very low signal, except for TRIM21 (Fig. 3b and Supplementary Fig. 4a) and LATS2 (clone No. 20, shown in purple), suggesting that the non-specific binding of AirID to human proteins is very low. The biotinylation of LATS2 by AirID alone was confirmed by AlphaScreen (Supplementary Fig. 5), which suggested that the AirID protein binds to LATS2. In the two assays using AirID-TP53 (Fig. 3e and Supplementary Fig. 4b) and AirID-IκBα (Fig. 3f and Supplementary Fig. 4c) fusion proteins, these proteins specifically biotinylated four (clone Nos. 12, 41, 44, and 78) and three (clone Nos. 53, 54, and 92) proteins, respectively, on the plate, underlined in red in Fig. 3c, d; clone No. 20 (LATS2) and the spot labelled T in Fig. 3c, d were from the cross-reaction described above. A comparison of the 1 × and 3 × spots of these biotinylated proteins indicated that the 1 × concentration was sufficient for detection (Fig. 3, and Supplementary Fig. 3c and 4). Of the proteins biotinylated by AirID-TP53, AURKA (STK15) (clone No. 12), TP53 (clone No. 44) and MDM2 (clone No. 78) proteins are known to interact with TP53^26,33–35^ and CREBBP (clone No. 41) were not previously known to interact with TP53. Of the proteins biotinylated by AirID-IκBα, the REL (clone No. 53) and RelA (clone No. 54) proteins are well known to interact with IκBα^36^ and EFHC2 (clone No. 92) was not previously known to interact with IκBα.Taken together, these results indicated that the CF-PPiD system works using the 1536-well formatted protein array with a wide variety of proteins.

### Screening of IκBα interacting proteins by CF-PPiD using a 20 K human protein beads array

Because CF-PPiD can be used to detect PPIs on a single 1536-well plate (Fig. 3 and Supplementary Fig. 4), we used a genome wide-scale protein array consisting of ~ 20,000 human recombinant proteins (20 K Human Protein Beads Array) to construct a method for biochemically direct interactome analysis. Human recombinant proteins from 19,712 cDNA templates were synthesized in 54 384-well plates using the wheat cell-free protein production system, and subsequently were spotted in 1536-well plates. Each protein at the 1 × amount described above was captured as a dual spot on the 1536-well formatted plate by glutathione-conjugated magnetic beads (Fig. 1a). Finally, a total of 19,712 human recombinant proteins were arrayed on 27 1536-well plates.

**Figure 4:**
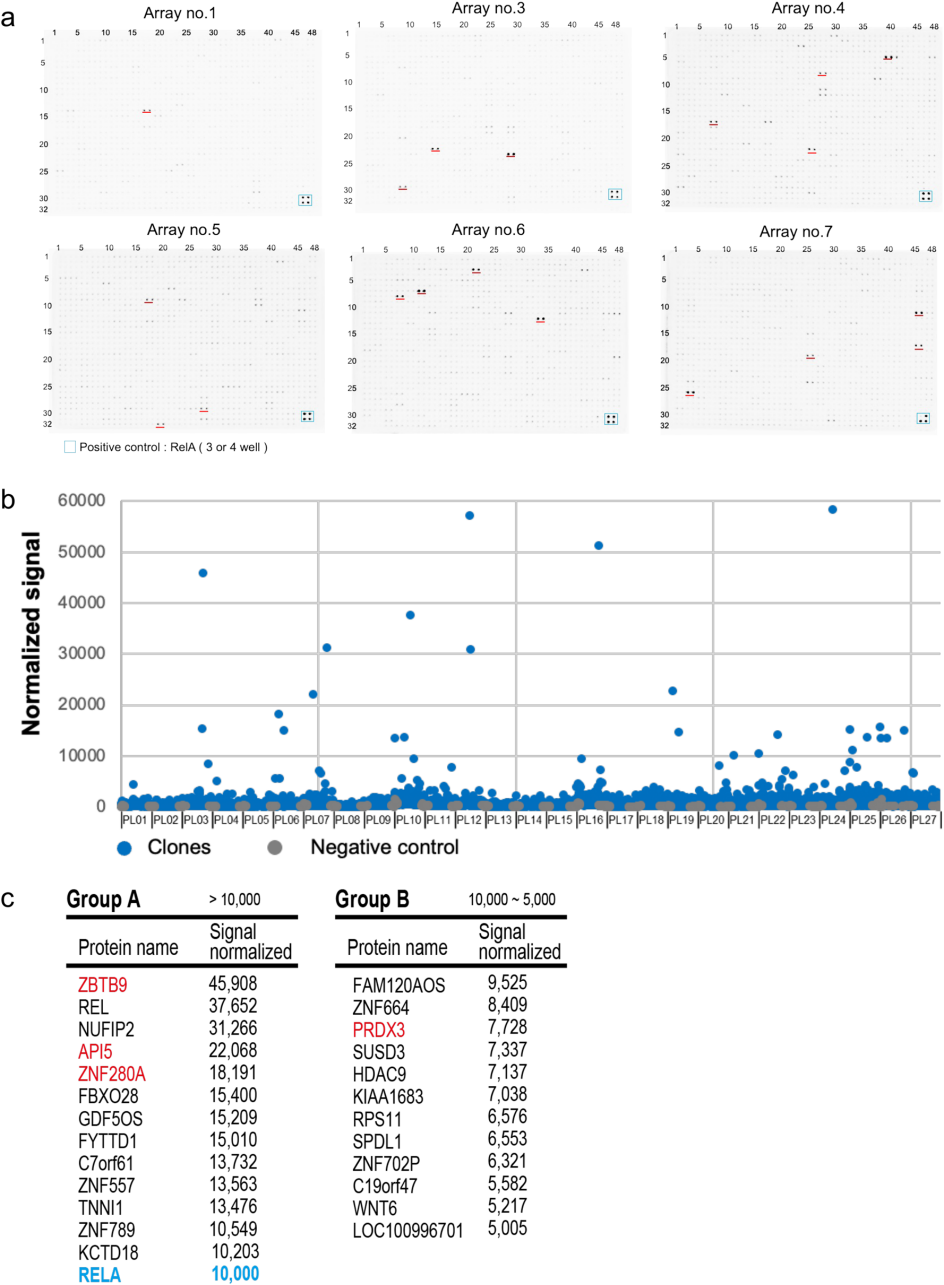
In vitro interactome analysis of IκBα using CF-PPiD. (**a**) Six panels were used for detection of biotinylated spots using AirID-IκBα in the CF-PPiD system. Positive spots are labelled in a red underline. Blue box indicates positive control spots (RelA). (**b**) The dot-blotting graph of signal intensity from biotinylated spots on all the panels. The signal was normalized by the signal from the positive control (RelA). (**c**) Clones that had high signals, Groups A and B. Red and blue characters denote clones used in Fig. 5 and a positive clone (RelA) used for normalization, respectively. Source data are provided as Supplementary Table 2.

For the in vitro direct interactome analysis using the 20 K human protein beads array and proximity biotinylation enzyme AirID, we selected IκBα as a target protein because the interaction of IκBα with the protein RelA is well known^36^ and the RelA–IκBα interaction was detected using the diversity protein array (Fig. 3f). AirID-IκBα was applied to 27 1536-well plates with biotin and ATP by syringe. Following the experimental procedure shown in Fig. 1a, the biotinylated proteins were detected as dual spots (Fig. 4a and Supplementary Fig. 6) and the immunoblotting signal was scanned (Supplementary Table 2). RelA protein was spotted on each plate as a positive control and the signals were normalized by the signal from the positive control (Fig. 4b). After the normalization, the average signal value from all spots was 478. From the data analysis, we categorized the signals into three groups: Group A had higher signals than the positive control RelA (> 10,000), Group B had signals between RelA and 50% of the RelA signal (5000 to 10,000), and Group C had signals between 50 and 20% of the RelA signal (2000 to 5000, more than four times higher than the average signal) (Fig. 4c and Supplementary Fig. 7). In the high signal clones, three carboxylase enzymes were found, pyruvate carboxylase (PC), methylcrotonyl-CoA carboxylase subunit 1 (MCCC1), and propionyl-CoA carboxylase subunit alpha (PCCA). These enzymes are known to be biotin-labelled proteins^37,38^, which indicated that these three carboxylase enzymes are background proteins in CF-PPiD and these three clones were excluded from the list. When these clones were excluded, the number of clones in Groups A, B, and C were 14, 12, and 106, respectively. The BioGRID database (https://thebiogrid.org/) was searched for reported interactions between these 132 proteins and IκBα. The analysis showed that 2, 1, and 3 clones were found in Groups A, B, and C, respectively (Supplementary Table 3).

We found 132 proteins as potential IκBα-binding partners using CF-PPiD, which were divided into Groups A, B, and C (Fig. 4c and Supplementary Fig. 7), and then these proteins were used for gene ontology (GO) analysis by Metascape (a gene annotation and analysis resource). According to the GO term and Metascape analyses, the genes interacting with IκBα in the PPI network were mainly related to ribosome, TNF-α/NF-κB signalling complex, regulation of protein catabolic process, base excision repair, intracellular protein transmembrane transport, Wnt ligand biogenesis and trafficking, and sensory organ development (Supplementary Fig. 8a and b). These results appear to be consistent with the fact that IκBα is known to be involved in TNF-α/NF-κB signalling^39,40^.

### In-cell analysis of IκBα interacting proteins found by CF-PPiD

**Figure 5:**
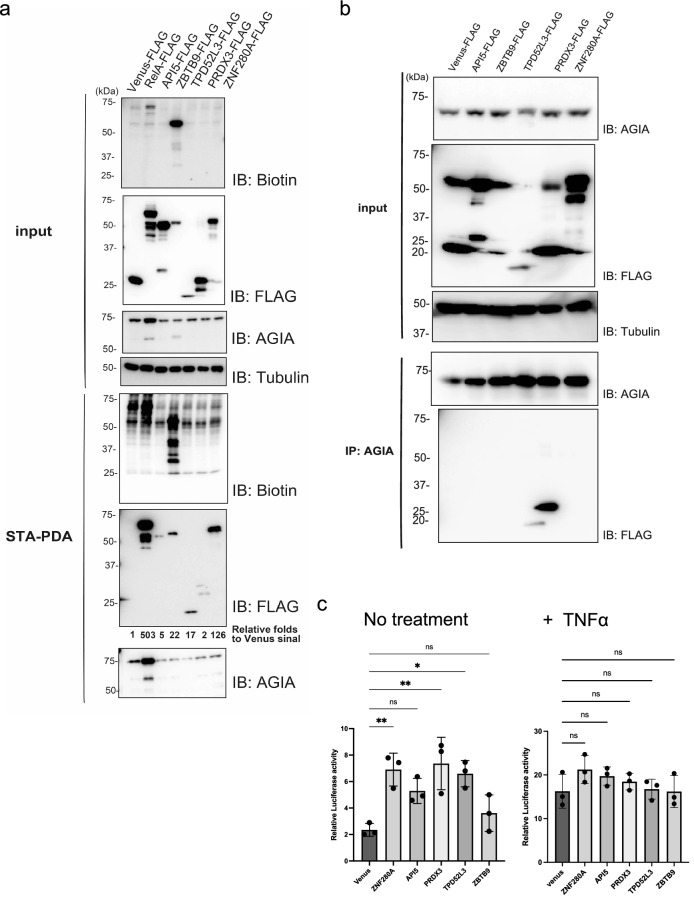
Validation of IκBα-interacting proteins in cells. (**a**) Streptavidin pull-down assay. The Venus gene, as a control, and each selected gene in pCAGGS-MCS-FLAG were transfected in HEK293T cells stably expressing AGIA-AirID-IκBα. After transfection for 24 h, d-biotin was added to 50 µM culture medium for 2 h. Then, the biotinylated proteins were pulled down using streptavidin beads and analysed by immunoblotting. (**b**) Co-immunoprecipitation. HEK293T cells stably expressing AGIA-AirID-IκBα were transfected with each selected gene in pCAGGS-MCS-FLAG. The control experiment used a transfected Venus gene. Immunoprecipitation was performed using anti-AGIA antibody. Immunoprecipitated proteins were detected with anti-FLAG and anti-AGIA antibodies. (**c**) Analysis of NF-κB activity by overexpression of each selected gene under basal conditions. HEK293T cells were transfected with each selected gene along with a luciferase reporter containing the NF-κB promoter, and then the cells were treated with DMSO (No treatment) or TNFα (+ TNFα) for 1 h. Data from each luciferase assay shown are derived from three independent experiments. Error bars denote the standard deviation (independent experiments: *n* = 3) and the *P*-values were calculated by one-way ANOVA with Tukey’s post-hoc test (**P = 0.002). Source data are provided as a Source data file.

CF-PPiD indicated the biotinylation of 132 clones in Groups A, B, and C (Fig. 4). To confirm the biotinylation of these proteins in cells, five clones, ZBTB9 (average signal (avs) 45,908), API5 (avs 22,068), ZNF280A (avs 18,191), PRDX3 (avs 7,728), and TPD52L3 (avs 2,040) were randomly selected from each group according to the signal. The genes for these proteins were inserted into a pCAGGS vector, and then transfected into HEK293T cells stably expressing AirID-IκBα^13^. The proteins were expressed and subjected to a pull-down assay with streptavidin beads (STA-PDA). To make clear the biotinylation in the STA-PDA, the band intensity (IB: FLAG) on the immunoblotting was scanned by ImageJ. All five clones showed more than two folds compared to Venus signal (negative control), indicating that all clones were biotinylated by AirID-IκBα stably expressed in the cells (Fig. 5a). Notably the biotinylation of ZBTB9 was found in “input” immunoblotting without STA-PDA, suggesting that ZBTB9 formed a stable complex with IκBα in the cells. Furthermore the cell lysates were immunoprecipitated by an anti-AGIA antibody^41^ because stably expressed AirID-IκBα has an *N*-terminal AGIA tag^13^. Interestingly, only the two proteins, PRDX3 and TPD52L3, were immunoprecipitated with AirID-IκBα (Fig. 5b) whereas ZBTB9 and ZNF280A that were strongly biotinylated were not immunoprecipitated. These results suggested that the enzymatic proximate biotinylation has a different sensitivity compared with immunoprecipitation for PPI analysis.

The RelA transcription factor induces NF-κB related genes^36,42^. We next investigated whether the expression of selected clones affected the RelA-transcriptional activity, with or without, TNF-α stimulation. To determine the RelA-transcriptional activity, each clone was transfected with two plasmids having the RelA gene and the NF-κB-promoter-luciferase gene in HEK293T cells and the luciferase activity was measured. Under the normal conditions without stimulation, the expression of four proteins (ZNF280A, API5, PRDX3, and TPD52L3) increased the RelA-transcriptional activity (Fig. 5c), and then the statistical test indicated the significance of three proteins on the transcriptional activity, whereas ZBTB9 expressions were no effect on the activity. In contrast, none of the selected proteins had any effect on the luciferase expression level under TNF-α stimulation. Because IκBα is known to be an inhibitor of the nuclear translocation of RelA in the normal condition and TNF-α stimulation induces the degradation of IκBα protein^36^, the enhancement of RelA-transcriptional activity by the addition of an IκBα-interacting protein without TNF-α stimulation appears to be a reasonable result. Taken together, these results indicated that CF-PPiD can facilitate the discovery of proteins that function in conjunction with a target protein in cells.

## Discussion

In the present study, CF-PPiD using a 1536-well format was developed for a biotinylation-based PPI assay. Using this format, the required amount of a target protein in a reaction mixture was a 1-mL translational mixture (approximately 50 µg of AirID-TP53 or 35 µg of AirID-IκBα) per plate without purification. Each protein was spotted at approximately 0.3 µL of translational mixture in a single spot. The amounts of all the reagents used were enough to be prepared in a standard laboratory. ELISA and AlphaScreen are also major candidate assays for large-scale PPI investigations^5,20,43,44^. However, in general, ELISA is not suitable for use for the PPI screening of approximately 20,000 proteins because the washing is a time-consuming step, and the assay requires purified recombinant proteins. The large-scale purification of recombinant proteins is also very time consuming and requires considerable refining. Because CF-PPiD involves performing GST-based purification on a magnetic plate, we directly used translational mixtures from the wheat cell-free system for both the target and spotting proteins. In addition, the washing during PPI screening is problematic because strong washing can eliminate weak PPIs and simple washing results in high non-specific background signals. In CF-PPiD, because the washing is carried out after the biotinylation as a PPI reaction, this method can detect a weak PPI. AlphaScreen technology is suitable for the large-scale investigation of PPIs because no protein purification or washing steps are required. We have previously used AlphaScreen technology for PPI screening^5,20,45,46^. However, the high cost of the AlphaScreen beads and a special device used for detection is a disadvantage with this method; for example, the cost of beads is more than US$ 8,000 for 20,000 assays even for an experimental design with *n* = 1. CF-PPiD is a highly specific, high-throughput, and inexpensive method for large-scale in vitro PPI analysis.

The proximity biotinylation enzyme AirID was used for PPI analysis using a protein array in this study. The assay flow is shown in Fig. 1a, when the AirID-fused protein interacts with a partner protein, AirID biotinylates the partner protein. After the biotinylation, the reaction mixture containing the AirID-fused proteins is washed out, and subsequently the biotin molecule on the partner protein is used for detection; the binding between the target protein and the interacting proteins is no longer required for the detection. In general, conventional in vitro methods for PPI analysis rely on using a target protein with a detectable tag, such as a peptide, protein, or fluorescent probe^47^. Furthermore, to reduce non-specific and background signals, conventional PPI methods, such as immunoprecipitation, require a strong washing step. It is practically impossible to determine washing conditions in which only specific bonds are retained. The methodological principle of such methods, therefore, means that strong interactions are predominant and weak interactions will be overlooked. However, because the enzymatic proximity biotinylation used in this study forms a strong covalent bound with lysine (Lys) residues on the target protein^13^, even vigorous washing is acceptable. Indeed, ZBTB9 was biotinylated both in in vitro CF-PPiD and in cells, but ZBTB9 was not immunoprecipitated (Fig. 4 and 5b, c). These results indicated that the proximity biotinylation results in the creation of a constant covalent biotin label on ZBTB9 lysine residues, enabling interaction detection with harsh washings. These results indicated that proximity biotinylation is suitable for the detection of PPIs.

CF-PPiD found 132 biotinylated proteins using AirID-IκBα. Approximately 19,712 human proteins were used for CF-PPiD, therefore, < 0.1% of the proteins on the array were biotinylated by AirID-IκBα. The signal range was from 2,009 to 51,356. Although the biotinylation signal of TPD52L3 was 2,040, it was a positive signal in both in-cell biotinylation and immunoprecipitation, indicating that lower signal clones, which showed approximately 20% of the signal of the positive clone (RelA), in Group C could also potentially be proteins that interact with IκBα in cells. Using these 132 biotinylated proteins, GO analysis by Metascape indicated that the IκBα protein was associated with six PPI networks, including ribosome, TNF-α/NF-κB signalling complex, base excision repair, and Wnt ligand biogenesis (Supplementary Fig. 8). IκBα binds to ribosome protein S3^48^ and crosstalk between NF-κB and Wnt/β-catenin signalling cascades has been suggested^49^. In addition, NF-κB signalling via IκBα is induced by DNA damage^50,51^. The biotinylated proteins found in this study provide specific research targets for these biological events.

Enzymatic proximity biotinylation occurs at Lys residues on the target protein^13,52^. Therefore, different numbers of surface Lys residues on a protein may result in different signals. Thus, the biotinylation signal from CF-PPiD would be affected, not only the strength of the PPI, but also by the number of Lys residues on the protein surface. The CF-PPiD system using the combination of proximity biotinylation and a protein array was the first trial of large-scale PPI analysis. Fine tuning of this method using proximity biotinylation will be the next challenge in our laboratory. In conclusion, CF-PPiD is a promising platform for the identification of target proteins and PPI analysis.

## Materials and methods

### Antibodies

The following HRP-conjugated antibodies were used in this study: FLAG (Sigma-Aldrich, A8592, MBL, M185-7), AGIA (produced in our laboratory)^41^, α-tubulin (MBL, PM054-7), and biotin (Cell Signaling Technology, #7075). To detect the proteins spotted on the array, anti-DDDDK-tag mAb-Alexa Fluor 647 (MBL, M185-A64) was used.

### Construction of the in vitro transcription templates

Each gene was selected from the cDNA library from the Functional Analysis of Protein and Research Application Project (NEDO, FLJ Human cDNA Database, http://flj.lifesciencedb.jp)^20,21^and then was amplified by PCR. The DNA fragments of the open-reading frame (ORF) were cloned in a pDONR201 vector using the gateway cloning system (Thermo Fisher Scientific). After confirming sequences, we generated the expression vectors by LR Clonase recombination with pEU-FLAG-GST-GW vectors for in vitro transcription. Then, the regions of the gene containing the ORF and tag sequence were amplified by PCR and used as transcription templates. The restriction enzyme sites were added to Venus, RelA, API5, ZBTB9, TPD52L3, PRDX3, and ZNF280A by PCR. Venus, RelA, API5, ZBTB9, TPD52L3, PRDX3, and ZNF280A were cloned into pCAGGS-MCS-FLAG.

For the diversity protein array, each gene was selected from the protein expression vector set which prepared with cDNA resources of the Kazusa DNA Research Institute that cloned into pEU-FLAG-GST vector using restriction enzymes for in vitro transcription^53^. The regions of the gene containing the ORF and tag sequence were amplified by PCR and used as transcription templates.

### Protein synthesis by a wheat cell-free protein production system

For construction of the protein array, proteins were synthesized in a well on a 384-well plate^21^. The FLAG-GST fusion human full-length proteins were synthesized using the WEPRO7240G Expression Kit (CellFree Sciences, Matsuyama, Japan). Translation reactions were performed using a bilayer method. For the bilayer system, 4.42 µL of reaction mixture containing 1.67 µL of WEPRO7240G wheat germ extract, 0.11 µL of RNase inhibitor, 2.5 µL of mRNA, and 0.14 µL of 20 mg/ml creatine kinase was overlaid with 45.58 µL of SUB-AMIX SGC (CellFree Sciences) in a 384-well titre-plate and incubated at 26 °C for 18 h. All dispensing processes for protein synthesis were carried out by a fully automatic dispenser (FUJIFILM Wako Pure Chemical Corporation).

### Construction of diversity protein array

Protein synthesis of 118 human full-length cDNA harbouring FLAG and GST (FG) tags was performed with the wheat cell-free protein expression system described above, and the synthesized proteins were absorbed onto the surface of glutathione-conjugated magnetic beads on an array plate (1,536 wells × 1 plate, Diversity Protein Array, CellFree Science), as follows. Magnetic beads with glutathione ligand were added to a translational mixture containing a synthesized protein bearing the FG tag, and the synthesized protein was absorbed onto the surface of the beads. Beads with adsorbed synthesized protein were dispensed on the array plate, which had a magnetic plate at the bottom. Each human protein was immobilized at the bottom of the array plate via the magnetic beads in the solution.

### Construction of 20 K human protein beads array

Protein synthesis of 19,712 human full-length cDNAs harbouring FG tags was performed with the wheat cell-free system described above, and the synthesized proteins were absorbed onto the surface of glutathione-conjugated magnetic beads on an array plate (1,536 wells × 27 plates, 20 K Human Protein Beads Array, CellFree Science, Matsuyama, Japan). Magnetic beads with glutathione ligand were added to a translational mixture containing a synthesized protein bearing the FG tag, and the synthesized protein was absorbed onto the surface of the beads. Beads with adsorbed synthesized protein were dispensed on the array plate, which had a magnetic plate at the bottom. Each human protein was immobilized at the bottom of the array plate via the magnetic beads in the solution.

### Biotinylation of the protein array

The arrays were blocked with 50 mM HEPES (pH 7.5), 200 mM NaCl, 0.08% Triton-X, 25% glycerol, 5 mM glutathione, and 0.3% skim milk. For biotinylation analysis by an AirID-fused protein, the arrays were incubated with a reaction mixture, consisting of a 1 mL translational mixture of each AirID-fused protein (50 µg of AirID-TP53 or 35 µg of AirID-IκBα per plate), 5 mM d-biotin and 1 mM ATP in PBS buffer containing 1 × Synthetic Block (Thermo Fisher Scientific Corp., Carlsbad, CA, USA) or 0.05% Tween20 and 1% skim milk at room temperature (approximately 25 °C) for 1–24 h (normally 3 h). After washing with PBS buffer containing 0.5 M NaCl and 1% Triton X-100, the arrays were incubated with HRP-conjugated anti-biotin antibody (1/200 dilution) in PBS buffer containing 1 × Synthetic Block at room temperature (approximately 25 °C) for 1 h. After washing with PBST buffer, a chemiluminescence reagent was used for detection of the binding signals using ImageQuant LAS4000. Array-Pro Analyzer (Nippon Roper Ltd.) was used for signal quantification. The original blots are presented in Supplementary Figs. 9, 10 and 13.

### Cell culture and transfection

HEK293T cells and HEK293T cells stably expressing AGIA-AirID-IκBα^13^ were cultured in Dulbecco’s Modified Eagle Medium (DMEM) (FUJIFILM Wako Pure Chemical Corporation) supplemented with 10% foetal bovine serum (FUJIFILM Wako Pure Chemical Corporation) and 100 units/mL penicillin and 100 mg/mL streptomycin) (Gibco) at 37 °C under 5% CO_2_. We confirmed that the cell line was free of mycoplasma contamination. HEK293T cells were transiently transfected using PEI MAX—transfection grade linear polyethylenimine hydrochloride (Polysciences).

### Streptavidin pull-down assay (STA-PDA)

To prepare the STA-PDA, HEK293T cells stably expressing AGIA-AirID- IκBα were cultured in a 10-cm dish. Each gene was transfected into cells. After incubation for 24 h, d-biotin was added to the cell culture (final concentration of 50 µM) and incubated at 37 °C for 2 h. The cells were harvested by suspension in TrypLE Select (Gibco). The cell pellets were washed with 1 mL of 1 × PBS buffer and lysed with 1 mL of lysis buffer [50 mM Tris–HCl (pH 7.5), 150 mM NaCl, and 1% sodium dodecyl sulfate (SDS)] containing a protease inhibitor cocktail, and the lysates were denatured by boiling at 98 °C for 15 min and sonication. Then, the lysates were clarified by centrifugation at 16,100 × *g* for 15 min. The cell lysates (970 µL) were added to 200 µL of lysis buffer containing 20 µL of streptavidin sepharose beads (GE Healthcare) and rotated at 27 °C for 1 h. After the flow-through solvent was removed, the beads were washed three times using 1 mL of wash buffer [50 mM Tris–HCl (pH 7.5), 1% SDS, and 150 mM NaCl], and the beads were boiled in 40 µL of SDS sample buffer containing 5% 2-mercaptoethanol. The boiled solution was analysed using SDS-PAGE and immunoblotting.

### Immunoblotting

Immunoblotting was carried out following standard protocols. Briefly, proteins were applied to SDS–polyacrylamide gel electrophoresis (SDS-PAGE) and transferred onto a polyvinylidene difluoride PVDF membranes. The membranes were blocked using 5% skim milk (Megmilk Snow Brand) in TBST buffer (20 mM Tris–HCl pH 7.5, 150 mM NaCl, 0.05% Tween20), and treated with the indicated antibodies and an HRP-conjugated antibody. Immobilon (Millipore) or ImmunoStar LD (FUJIFILM Wako Pure Chemical Corporation) were used as the substrate for HRP, and the luminescence signal was detected using an ImageQuant LAS 4000 mini (GE Healthcare). In some blots, the membrane was stripped with stripping solution (FUJIFILM Wako Pure Chemical Corporation) and re-probed with other antibodies. All immunoblot data and band intensities were analysed using ImageJ software. The original blots are presented in Supplementary Figs. 11 and 12.

### Immunoprecipitation

Each gene was transfected to cells stably expressing AGIA-AirID-IκBα that were cultured in a 10-cm dish. After incubation for 24 h, the cells were harvested by suspension in TrypLE Select (Gibco). The cell pellets were washed with 1 mL of 1 × PBS buffer and lysed with 1 mL of IP lysis buffer (25 mM Tris–HCl pH 7.5, 150 mM NaCl, 1 mM EDTA, 1% NP-40, and 5% glycerol) with protease inhibitors (Roche) and the lysates were rotated at 4 °C for 30 min. Then, 970 µL of the lysate was added to 50 µL of IP lysis buffer containing 10 µL of protein A Dynabeads (Thermo Fisher Scientific) and rotated at 4 °C for 30 min. After the rotation at 4 °C, the supernatant was recovered. For immunoprecipitation, the supernatant was added to 1 µg of the anti-AGIA antibodies and rotated at 4 °C for 2 h. Then, the supernatant was added to 50 µL of IP lysis buffer containing 10 µL of protein A Dynabeads (Thermo Fisher Scientific) and rotated at 4 °C overnight. After washing two times with 1 mL of 1 × PBS and once time with 1 mL of IP lysis buffer, the immunocomplexes were boiled in 40 µL of SDS sample buffer containing 5% 2-mercaptoethanol. The boiled solution was analysed using SDS-PAGE and immunoblotting.

### In vitro biotinylation assay using AlphaScreen technology

AGIA-AirID was added to FG-DHFR or FG-LATS2. In addition, d-biotin (final concentration 500 nM) and NaCl (final concentration 100 mM) were added to the reaction mixture and incubated at 37 °C for 3 h. Biotinylation was detected by using the AlphaScreen IgG (Protein A) detection kit (Perkin Elmer). Briefly, 25 μL of detection mixture containing 1 µL of the reaction mixture, 100 mM Tris–HCl (pH 8.0), 0.1% Tween 20, 100 mM NaCl, 10 ng of anti-FLAG antibody (Sigma), 1 mg/mL BSA, 0.08 μL of streptavidin-coated donor beads, and 0.08 μL of protein A-conjugated acceptor beads were added to each well of a 384-well Alphaplate before incubation at 26 °C for 1 h. Luminescence signal was detected using the AlphaScreen detection program with an EnVision device (PerkinElmer).

### Dual-luciferase reporter assays

All reporter assays were performed using a Dual-Luciferase Assay Kit (Promega). HEK293T cells were transfected with pGL4.32 [luc2P/NF-κB-RE/Hygro] and reporters together with the pRL-TK reporter. The cells were treated with TNF-α (20 ng/ml) at 1 h, or not treated, 20 h after transfection.

### Statistical analysis and reproducibility

The data are shown as the means ± standard deviation (SD) from the more than three technical replicates. Significant changes were analysed by one-way analysis of variants (ANOVA) followed by Tukey's tests using GraphPad Prism (version 9) software (GraphPad, Inc.)

## Supplementary Information

Supplementary Figures.

Supplementary Figures.

Supplementary Table 1.

Supplementary Table 2.

Supplementary Table 3.

## Data Availability

Methods were performed in accordance to the relevant guidelines and regulation. The datasets generated and/or analysed during the current study are available in the CellFree Sciences repository, https://www.cfsciences.com/images/support_data/AirID_I_by_CFPPiD.pdf.
