# Supplementary material for: CF-PPiD technology based on cell-free protein array and proximity biotinylation enzyme for in vitro direct interactome analysis
Source: Sci Rep. 2022 Jun 22;12:10592. doi: 10.1038/s41598-022-14872-w (PMC9217950; doi:10.1038/s41598-022-14872-w)
Supplement: Supplementary file 2 — Supplementary Figures. [file 41598_2022_14872_MOESM2_ESM.pdf]

CF-PPiD technology based on cell-free protein array and proximity biotinylation enzyme for *in vitro* direct interactome analysis

Shusei Sugiyama[1#], Kohdai Yamada[2#], Miwako Denda[1], Satoshi Yamanaka[2], Satoshi Ozawa[1], Ryo Morishita[1*], Tatsuya Sawasaki[2*]

[1]*CellFree Sciences. Co. Ltd., 3 Bunkyo-cho, Matsuyama, Ehime 790-8577, Japan,* [2]*Proteo-Science Center, 3 Bunkyo-cho, Matsuyama, Ehime 790-8577, Japan*

[#] These authors contributed equally: Shusei Sugiyama, Kohdai Yamada.

[#] These authors contributed equally: Shusei Sugiyama, Kohdai Yamada.

*Corresponding Authors:

Ryo Morishita

CellFree Sciences. Co. Ltd., Matsuyama 790-8577, Japan

Tel: 81-89-925-1088, *E-mail:* rmorishita@cfsciences.com

Tatsuya Sawasaki

Proteo-Science Center, Ehime University, Matsuyama 790-8577, Japan

Tel: 81-89-927-8530, *E-mail:* sawasaki@ehime-u.ac.jp

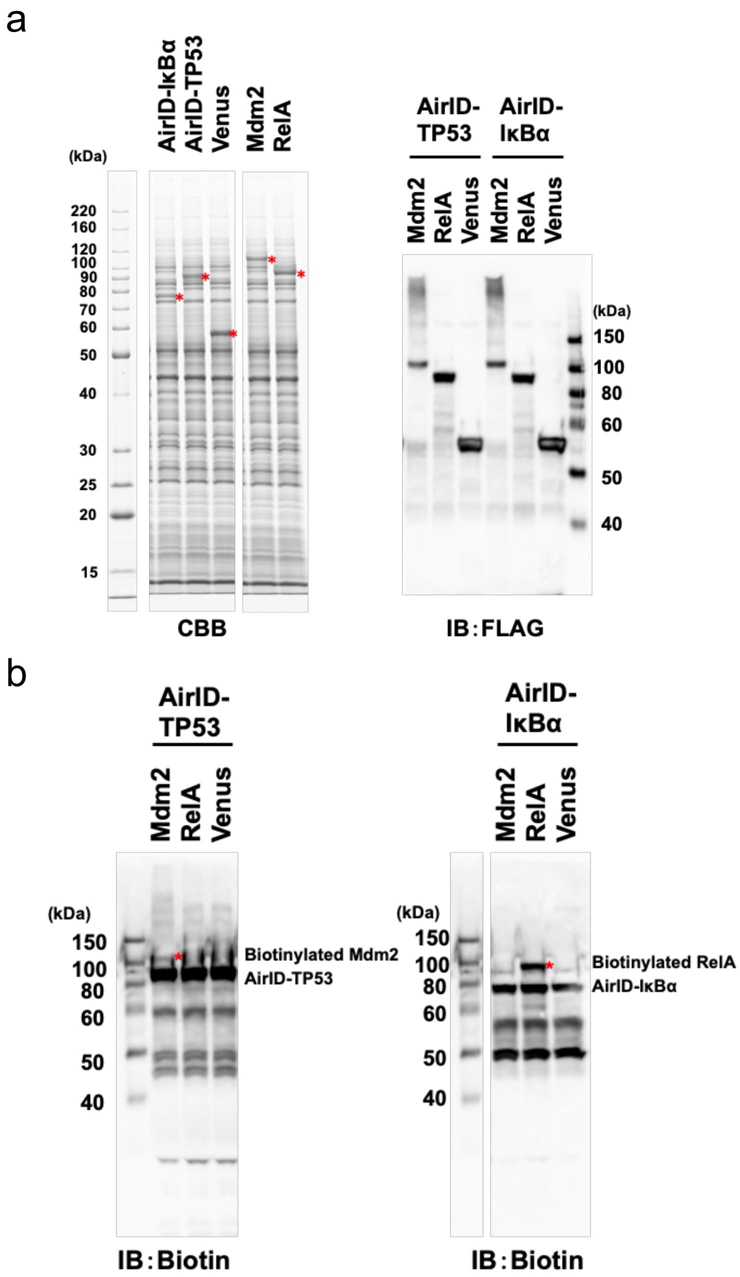

Supplementary Fig. 1. *In vitro* biotinylation assay of AirID-TP53 and AirID-IκBα.

a, Production of AirID-fused proteins and substrate proteins by a wheat cell-free protein production system. *N*-terminal AirID-fused proteins (AirID-TP53 and AirID-IκBα) and FG (FLAG-GST)-tagged substrate proteins (FG-RelA, FG-Mdm2, and FG-Venus) were synthesized using a wheat cell-free protein production system. The identity of the synthesized proteins was confirmed using coomassie brilliant blue (CBB) staining (red asterisk on left panel). The identity of the FG-tagged substrate proteins was confirmed using anti-FLAG antibody immunoblotting (right panel). Mixed AirID-fused proteins were not detected. **b,** Biochemical biotinylation of substrates by AirID-fused proteins. The biotinylation of FG-tagged proteins by the AirID-fused proteins in the solution containing AirID-fused protein, FG-tagged protein, biotin, and ATP was investigated using anti-biotin antibody immunoblotting. AirID-TP53 and AirID-IκBα biotinylated the respective partner proteins (red asterisk) but not any other proteins.

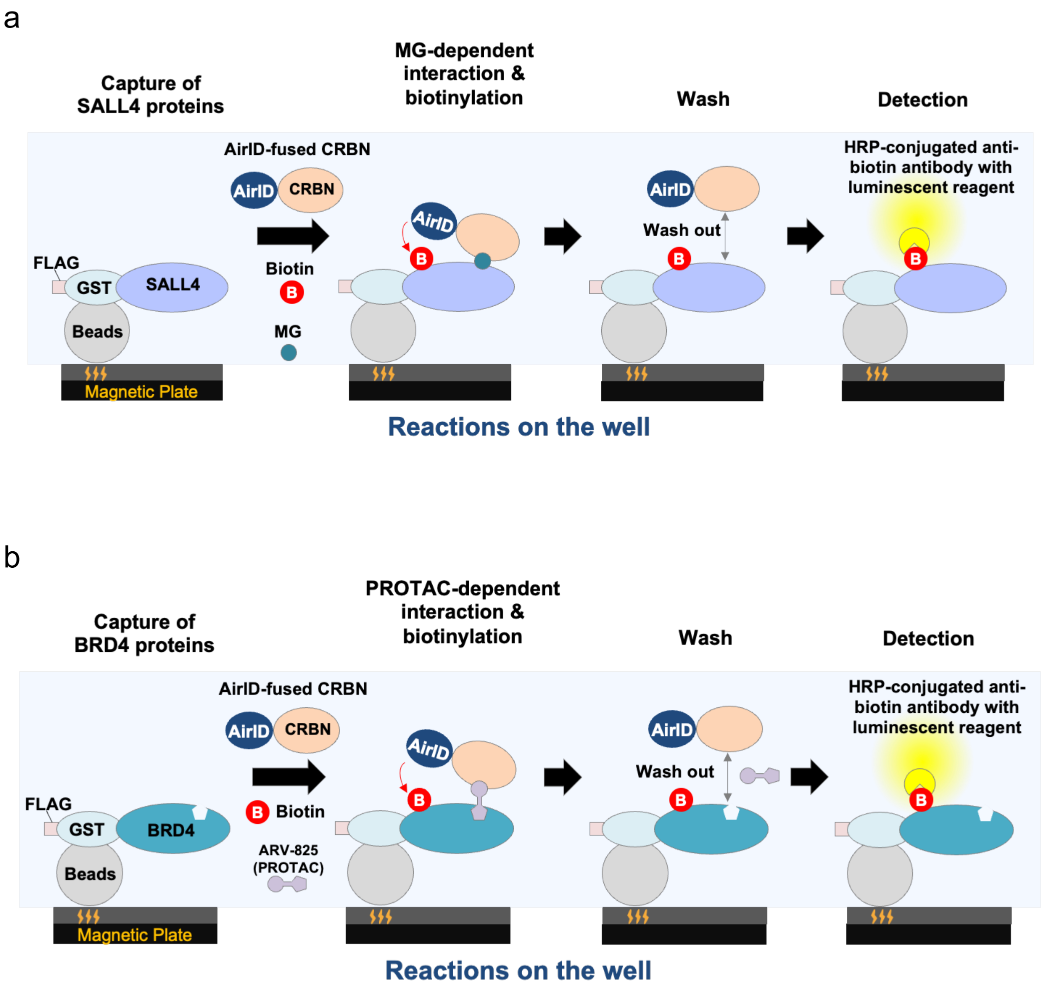

Supplementary Fig. 2. Scheme of the assay using CF-PPiD for molecular glue- and PROTAC-dependent biotinylation.

The reactions on the well of (**a**) molecular glue (MG)- or (**b**) PROTAC-dependent interactions using AirID-CRBN fusion protein with the CF-PPiD system. MG or PROTAC was added with an AirID-fused target protein, biotin, and ATP to the array. The rest of the procedure was the same as for standard PPIs.

a

Category	No. of Clone	Note
Protein kinase	21	3 clones are also categolized as epigenetics
PPase	14	
Epigenetics	16	4 clones are also categolized as DNA Binding
		1 clone is also categolized as E3 ligase
		3 clones are also categolized as protein kinase
Transcription factor	25	3 clones are also categolized as Epigenetics
		2 clones are also categolized as E3 ligase
E3 ligase	22	3 clones are also categolized as DNA Binding
		1 clone is also categolized as Epigenetics
Chaperone	2	
Uncategorized	19	
DUB	15	

b

c

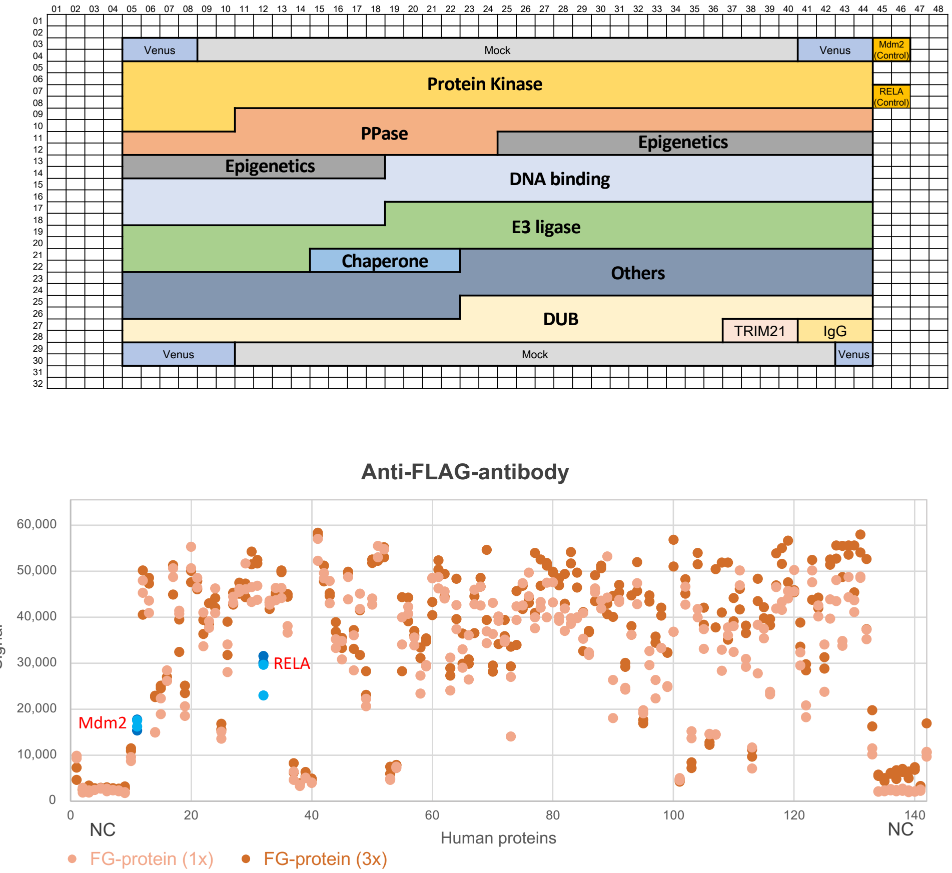

Supplementary Fig. 3. Proteins and detection of spotted proteins on the diversity protein array.

a Annotated protein categories on the diversity protein array. **b** Arrangement of categorized proteins on the diversity protein array. Selected proteins for the diversity protein array were arranged by category and immobilized on a 1536-well plate. Control proteins, including Venus, TRIM21, and IgG, were also spotted on the array. **c** The dot-blotting graph from the scanned image of spotted proteins on the diversity protein array detected with fluorescently labelled anti-FLAG antibody. The signal intensities of each protein spot on the array were calculated using image analysis software and plotted. Light orange dots represent the intensity of 1× beads spots, and dark orange dots represent 3× beads spots. Source data are provided as a Source data file and Supplementary Table_1.

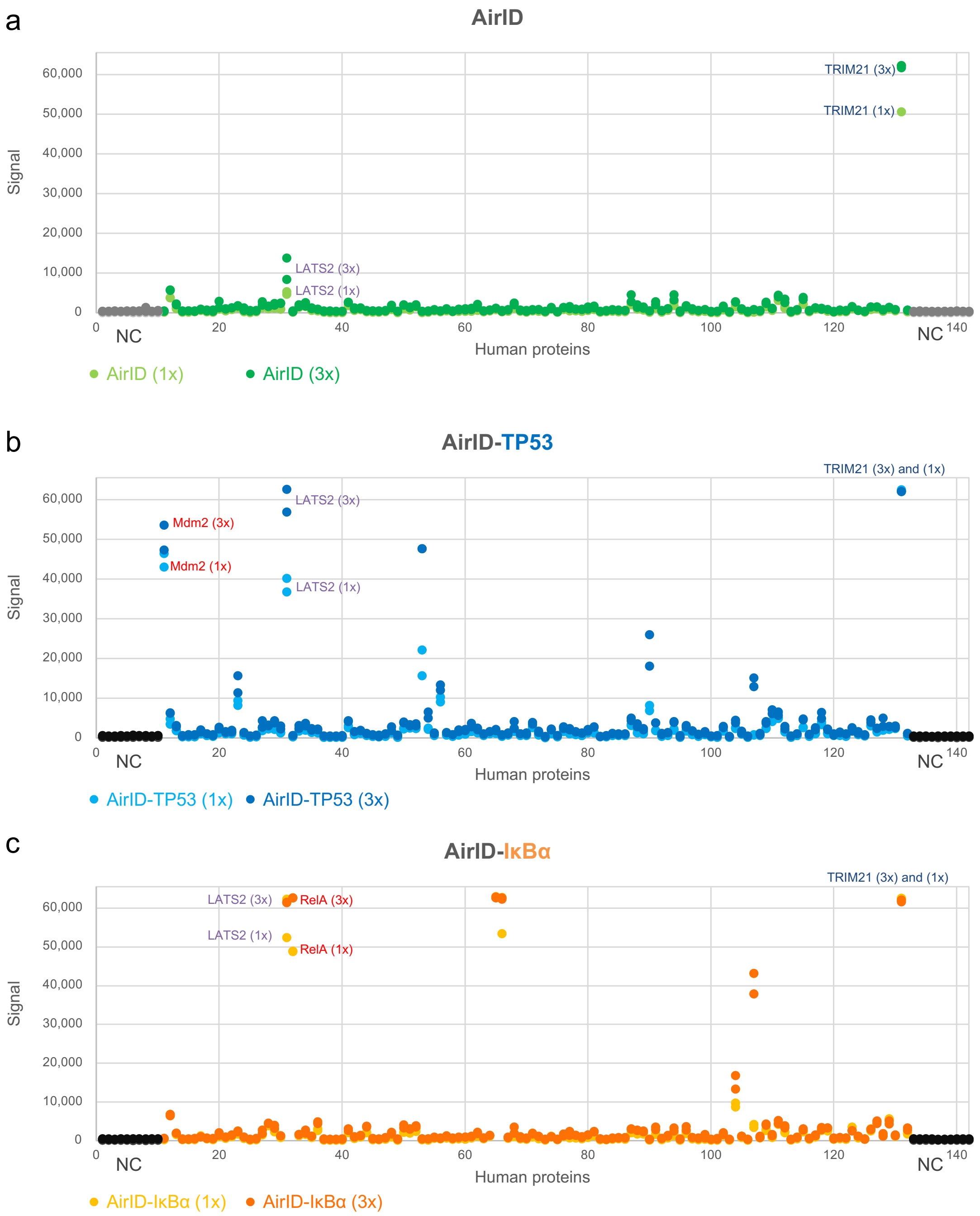

Supplementary Fig. 4. The dot-blotting graph from the scanned image of the PPI screening of AirID, AirID-TP53, and AirID-IκBα using the diversity protein array.

a, The dot-blotting graph from scan data of the PPI screening using AirID protein (Fig. 3b). **b,** The dot-blotting graph from scan data of the PPI screening using AirID-TP53 fusion protein (Fig. 3c). **c,** The dot-blotting graph from scan data of the PPI screening using AirID-IκBα fusion protein (Fig. 3d). In each graph, light coloured dots represent the signal intensity of 1× beads spots, and dark coloured dots represent the signal intensity of 3× beads spots. Grey to black dots represent negative control (mock or Venus) spots. Positive control sample spots are labelled in red. The spots of TRIM21, which interacted with antibody IgG, are labelled in dark blue, and the spots of LATS2, which interacted with AirID alone, are labelled in purple. Source data are provided as a Source data file and Supplementary Table 1.

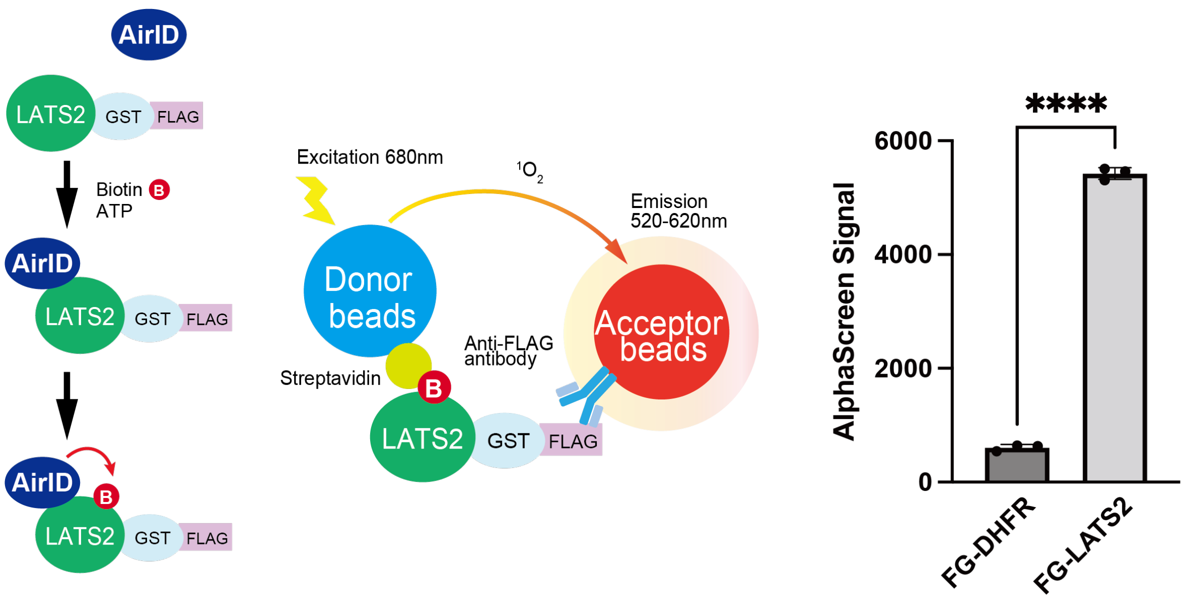

Supplementary Fig. 5. Biotinylation of LATS2 by AirID.

Using the translational mixtures from the wheat cell-free system, the biotinylation of FG-LATS2 was carried out by mixing FG-LATS2 with AirID, biotin, and ATP. The biotinylation was detected using AlphaScreen (right panel). Error bars denote the standard deviation (independent experiments: n = 3) and the *P*-values were calculated by one-way ANOVA with Tukey's post-hoc test (*****P* = 0.00001). Source data are provided as a Source data file.

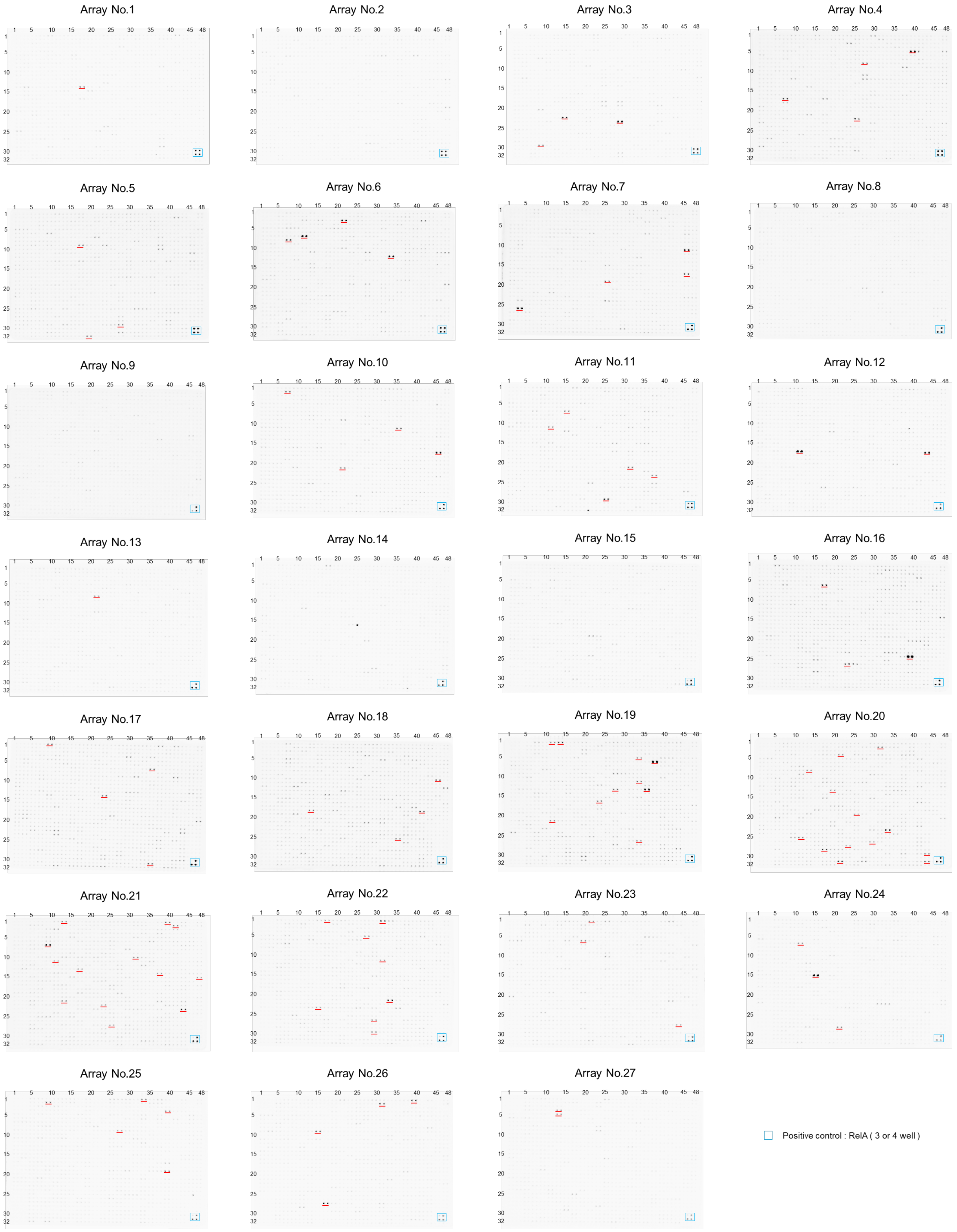

Supplementary Fig. 6. All panels from CF-PPiD using AirID-IκBα.

Positive spots are labelled in a red underline. Blue box indicates positive control spots (RelA).

Group C 5,000 ~ 2,000

5000 ~ 2500

Protein name	Signal normalized
BTRC	4855
RPS17	4773
RPL21	4597
SNX22	4542
MRPS18B	4531
PRR24	4244
RPL23	4035
RSL24D1	3998
LINC00602	3962
TSGA10IP	3940
EBAG9	3921
MLPH	3895
TMEM14A	3724
RPS9	3699
MEF2B	3445
C16orf59	3404
PPL	3350
ZNF833P	3334
LOC100996713	3287
B9D1	3273
TNNI2	3217
ATP13A1	3200
HOXA10	3122
HIST1H2BG	3116
DLX6	3094
ZNF574	3063
CCL11	3011
RPL38	2995
C11orf87	2994
C21orf67	2937
PITX2	2860
CSNK1E	2859
SPATA17	2850
ZNHIT1	2823
PROX2	2796
CCDC121	2785
FGFRL1	2773
MRPL20	2740
MRPL30	2732
RPS5	2697
PEX10	2684
NTHL1	2659
PFKFB4	2646
HHLA3	2612
ISCA1	2596
FAM211B	2591
MPLKIP	2589
UBXN1	2578
RAB36	2558
RNF217	2556
LETMD1	2550
HCLS1	2548
THAP6	2542
C8orf46	2538
PITX2	2506

2500 ~ 2000

Protein name	Signal normalized
MBD3L2	2498
RPL27A	2489
RPS16	2478
PHF21B	2464
POLD3	2450
SEC61B	2419
HIST1H2AC	2396
TMEM105	2387
NDUFB10	2382
WNT10A	2371
DNAJC19	2365
C19orf60	2365
H2AFV	2350
FAM83F	2350
WNT10B	2283
COX6A2	2279
LOC100132272	2278
ZNF645	2277
ZC2HC1C	2275
HNRNPA1	2266
GOLGA8G	2262
HIGD2A	2242
NBPF6	2233
PDGFA	2210
LINC00173	2203
FLJ36116	2202
C16orf11	2198
HGS	2190
HS3ST2	2190
LINC00305	2189
LIME1	2184
SGOL1	2179
LOC100996602	2169
WRAP53	2167
LIN37	2165
PINLYP	2154
EIF4E1B	2143
LARP4	2128
RAB26	2125
ZNF841	2116
LOC101060615	2096
VAX1	2074
PTRH1	2063
LOC100996325	2056
TPGS1	2047
RASA4	2045
TPD52L3	2040
TIMM23	2033
CISD2	2032
FSIP2	2018
FANCD2OS	2009

Supplementary Fig. 7. A list of Group C clones

Group C clones, which had normalized signals from 2,000 to 5,000. A red character denotes a clone used in Fig. 5.

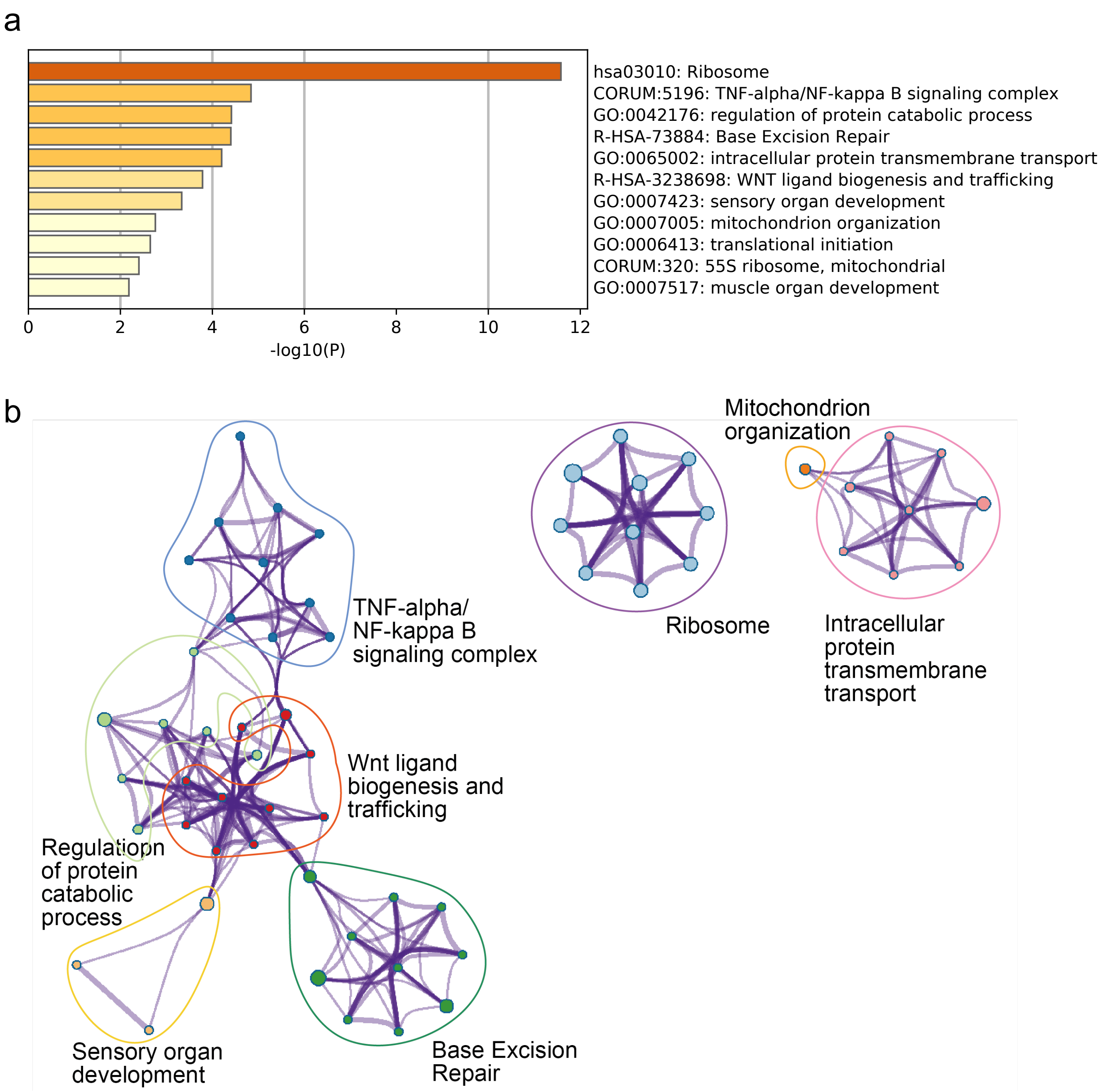

Supplementary Fig. 8. Gene ontology and Pathway analyses which had normalized signals from 2,000 to 5,000 IκBα.

a, Gene ontology analysis of IκBα interactome. Gene ontology analysis of IκBα-interacting proteins using CF-PPiD. IκBα-interacting proteins were selected with a normalized signal value that was > 2,000 (Groups A, B, and C). The gene ontology software Metascape (a gene annotation and analysis resource) was used to analyse protein interactions (https://metascape.org/gp/). **b,** Pathway analysis of IκBα interactome. Pathway analysis of IκBα-interacting proteins using CF-PPiD. IκBα-interacting proteins were selected that had a normalized signal value that was > 2,000 (Groups A, B, and C). The gene ontology software Metascape was used to analyse protein interactions (https://metascape.org/gp/).

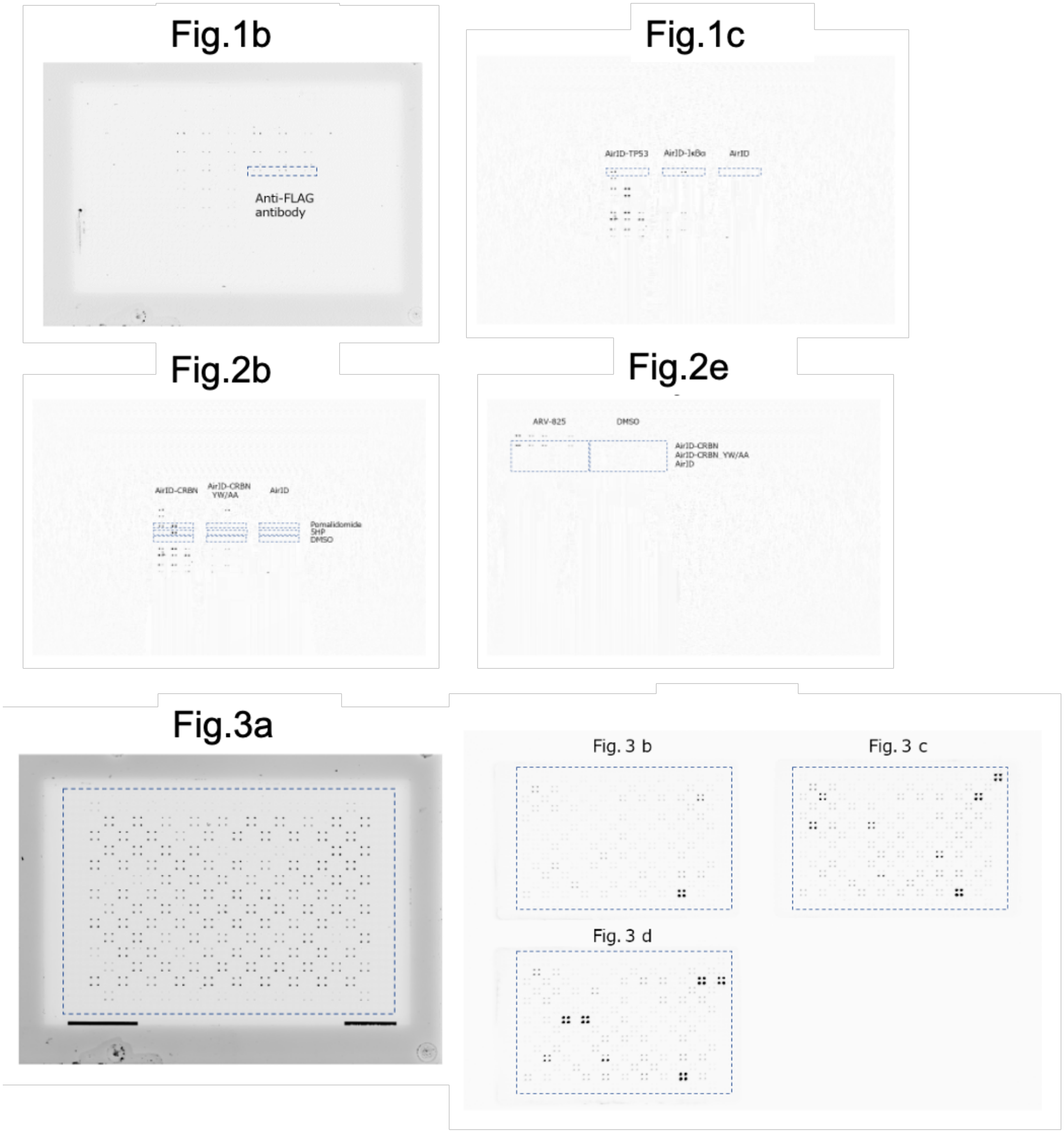

Supplementary Fig. 9. Full blot images of Western blotting assay (Fig. 1-3)

All blots were performed using ImageJ.

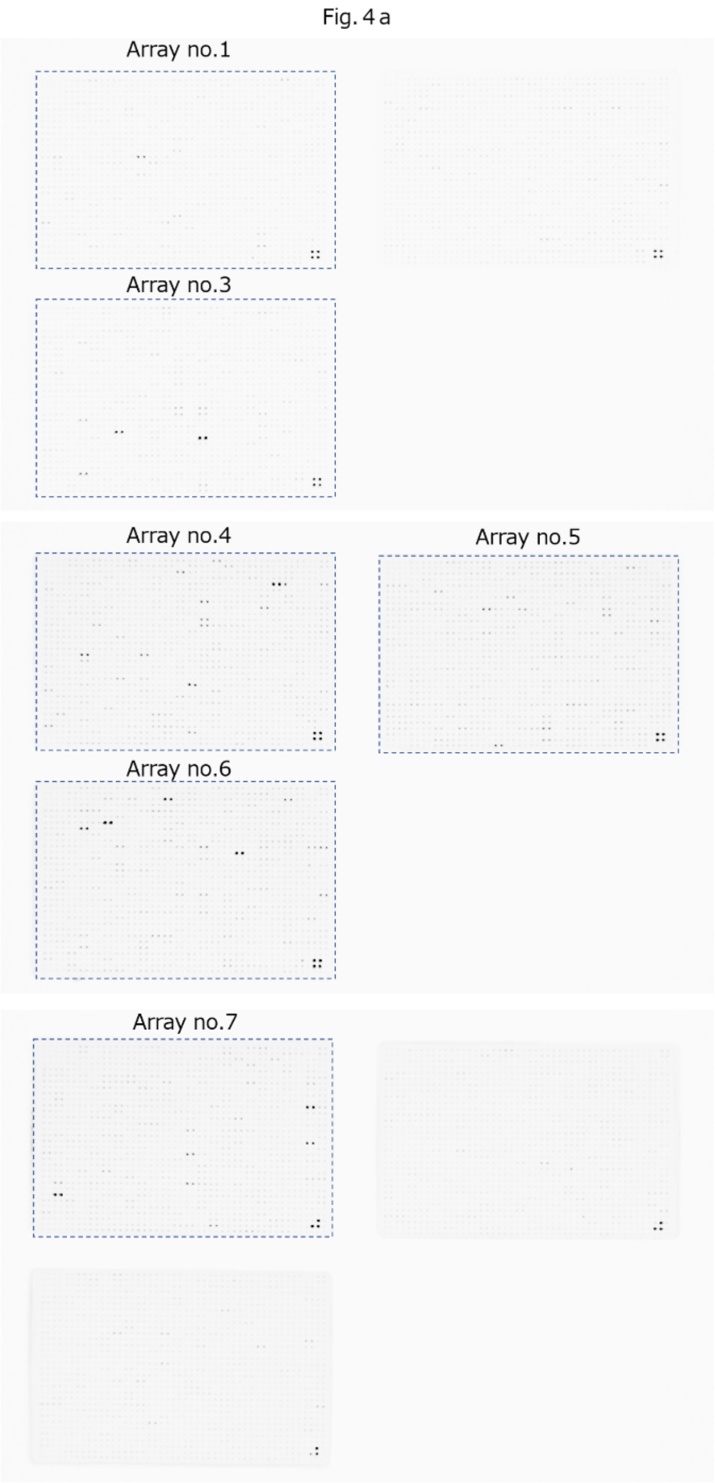

Supplementary Fig. 10. Full blot images of Western blotting assay (Fig. 4)

All blots were performed using ImageJ.

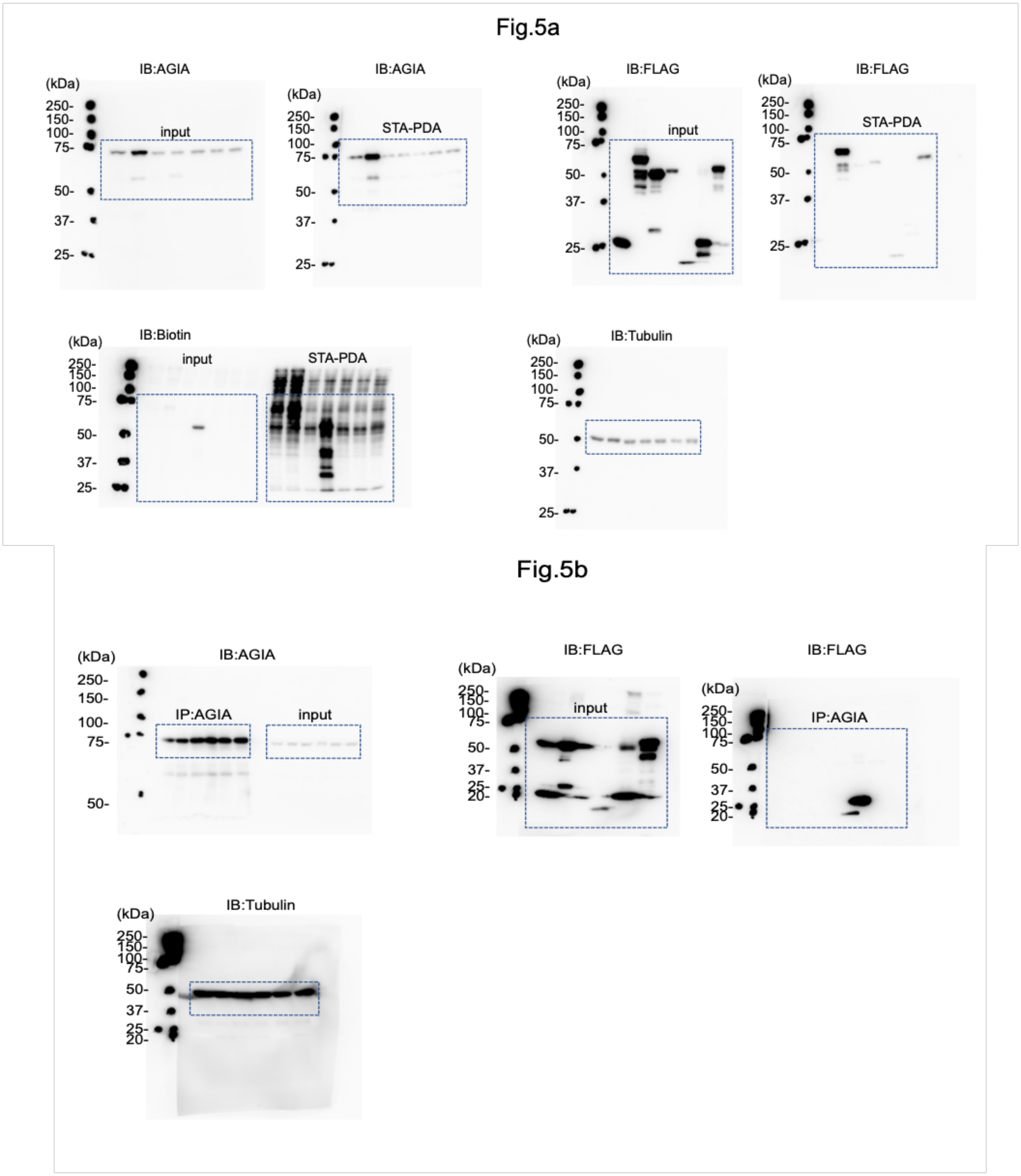

Supplementary Fig. 11. Full blot images of Western blotting assay (Fig. 5)

All blots were performed using ImageJ.

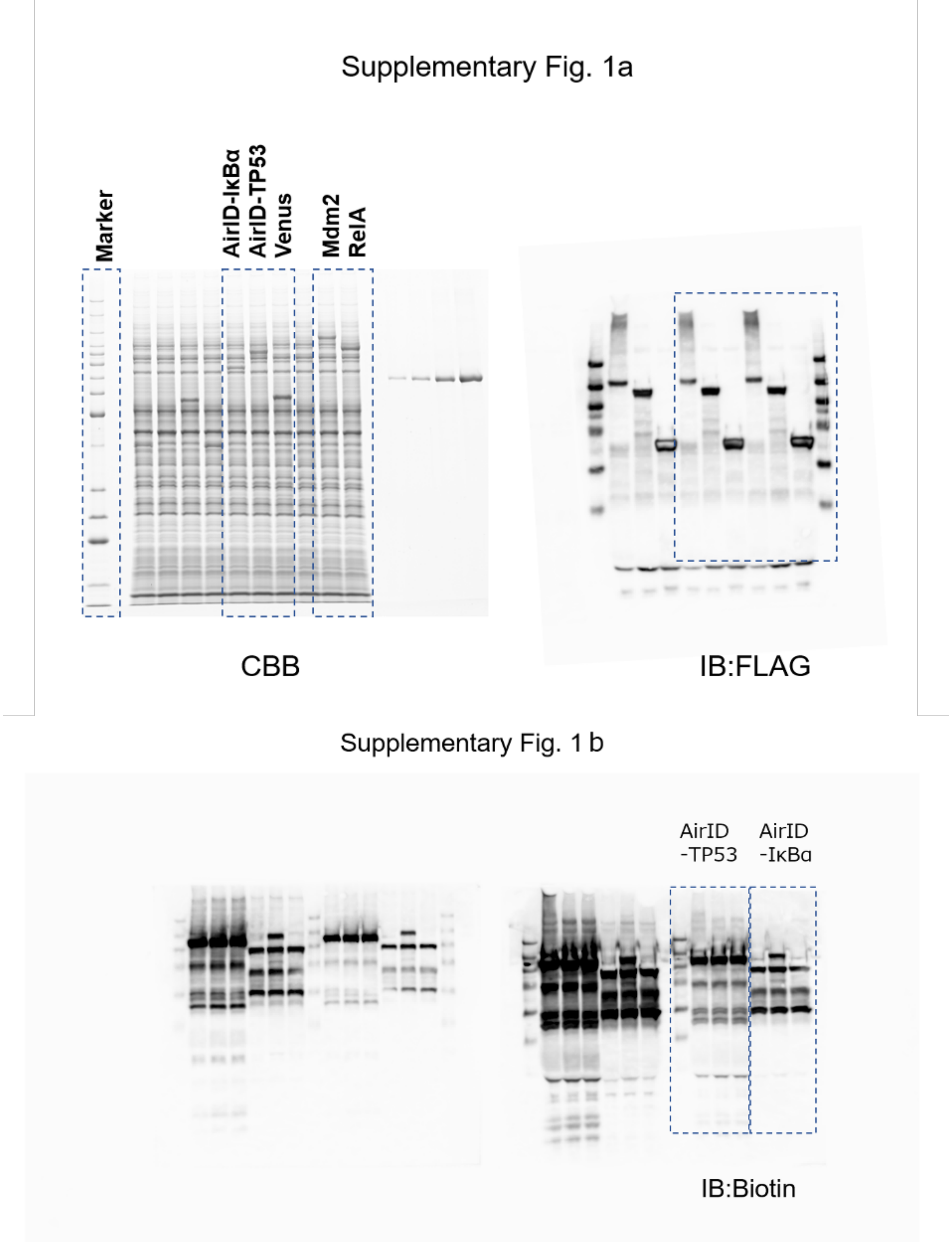

Supplementary Fig. 12. Full blot images of Western blotting assay (Supplementary Fig.1)

All blots were performed using ImageJ.

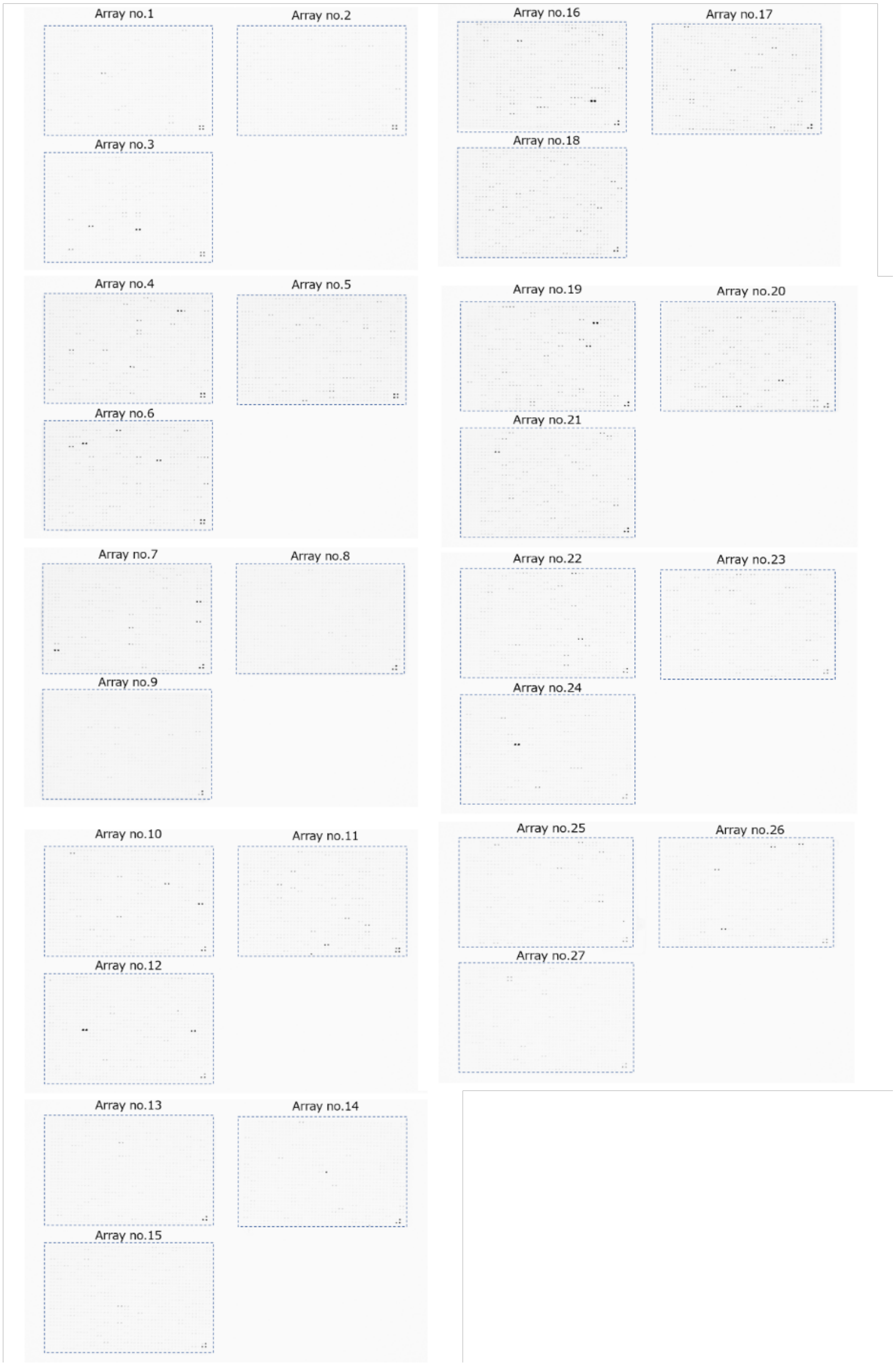

Supplementary Fig. 13. Full blot images of Western blotting assay (Supplementary Fig.6)

All blots were performed using ImageJ.
